# Carotid plaque macrophage burden and inflammatory lipid-associated macrophage markers predict secondary major adverse cardiovascular events after endarterectomy

**DOI:** 10.1093/eurheartj/ehag117

**Published:** 2026-02-27

**Authors:** Koen H M Prange, Gemma Bel-Bordes, Marie A C Depuydt, Panos Barlampas, Moritz J Reif, Max L B Grönloh, Rosalie W M Kempkes, Guillermo R Griffith, Cindy van Roomen, Yayuan Zhu, Andreas Edsfeldt, Jiangming Sun, Maaike J M de Jong, Barend M Mol, Bram Slütter, Ilze Bot, Annette E Neele, Dominique P V de Kleijn, Gert J de Borst, Jeffrey Kroon, Anouk Wezel, Harm J Smeets, Erik S G Stroes, Jaap D van Buul, Pieter Goossens, Johan Kuiper, Isabel Goncalves, Gerard Pasterkamp, Michal Mokry, Menno P J de Winther

**Affiliations:** Department of Medical Biochemistry, Amsterdam UMC, University of Amsterdam, Meibergdreef 9, 1105 AZ, Amsterdam, The Netherlands; Amsterdam Cardiovascular Sciences (ACS), Atherosclerosis & Ischemic Syndromes, Amsterdam UMC, Meibergdreef 9, 1105 AZ, Amsterdam, The Netherlands; Amsterdam Institute for Immunology and Infectious Diseases (AII), Inflammatory Diseases, Amsterdam UMC, Meibergdreef 9, 1105 AZ, Amsterdam, The Netherlands; Laboratory of Clinical Chemistry and Haematology, University Medical Center, Heidelberglaan 100, 3584 CX, Utrecht, The Netherlands; Division of BioTherapeutics, Leiden Academic Centre for Drug Research, Leiden University, Leiden, The Netherlands; Cardiovascular Research–Translational Studies, Clinical Sciences Malmö, Lund, Lund University, Sweden; Department of Medical Biochemistry, Amsterdam UMC, University of Amsterdam, Meibergdreef 9, 1105 AZ, Amsterdam, The Netherlands; Amsterdam Cardiovascular Sciences (ACS), Atherosclerosis & Ischemic Syndromes, Amsterdam UMC, Meibergdreef 9, 1105 AZ, Amsterdam, The Netherlands; Amsterdam Institute for Immunology and Infectious Diseases (AII), Inflammatory Diseases, Amsterdam UMC, Meibergdreef 9, 1105 AZ, Amsterdam, The Netherlands; Department of Pathology, Maastricht University Medical Centre+, Maastricht University, Maastricht, The Netherlands; Cardiovascular Research Institute Maastricht (CARIM), Maastricht University, Maastricht, The Netherlands; Department of Medical Biochemistry, Amsterdam UMC, University of Amsterdam, Meibergdreef 9, 1105 AZ, Amsterdam, The Netherlands; Amsterdam Cardiovascular Sciences (ACS), Atherosclerosis & Ischemic Syndromes, Amsterdam UMC, Meibergdreef 9, 1105 AZ, Amsterdam, The Netherlands; Amsterdam Institute for Immunology and Infectious Diseases (AII), Inflammatory Diseases, Amsterdam UMC, Meibergdreef 9, 1105 AZ, Amsterdam, The Netherlands; Department of Medical Biochemistry, Amsterdam UMC, University of Amsterdam, Meibergdreef 9, 1105 AZ, Amsterdam, The Netherlands; Amsterdam Cardiovascular Sciences (ACS), Atherosclerosis & Ischemic Syndromes, Amsterdam UMC, Meibergdreef 9, 1105 AZ, Amsterdam, The Netherlands; Amsterdam Institute for Immunology and Infectious Diseases (AII), Inflammatory Diseases, Amsterdam UMC, Meibergdreef 9, 1105 AZ, Amsterdam, The Netherlands; Department of Medical Biochemistry, Amsterdam UMC, University of Amsterdam, Meibergdreef 9, 1105 AZ, Amsterdam, The Netherlands; Amsterdam Cardiovascular Sciences (ACS), Atherosclerosis & Ischemic Syndromes, Amsterdam UMC, Meibergdreef 9, 1105 AZ, Amsterdam, The Netherlands; Amsterdam Institute for Immunology and Infectious Diseases (AII), Inflammatory Diseases, Amsterdam UMC, Meibergdreef 9, 1105 AZ, Amsterdam, The Netherlands; Department of Medical Biochemistry, Amsterdam UMC, University of Amsterdam, Meibergdreef 9, 1105 AZ, Amsterdam, The Netherlands; Amsterdam Cardiovascular Sciences (ACS), Atherosclerosis & Ischemic Syndromes, Amsterdam UMC, Meibergdreef 9, 1105 AZ, Amsterdam, The Netherlands; Amsterdam Institute for Immunology and Infectious Diseases (AII), Inflammatory Diseases, Amsterdam UMC, Meibergdreef 9, 1105 AZ, Amsterdam, The Netherlands; Laboratory of Clinical Chemistry and Haematology, University Medical Center, Heidelberglaan 100, 3584 CX, Utrecht, The Netherlands; Experimental Cardiology, Department of Heart and Lungs, University Medical Centre Utrecht, Heidelberglaan 100, 3584 CX, Utrecht, The Netherlands; Cardiovascular Research–Translational Studies, Clinical Sciences Malmö, Lund, Lund University, Sweden; Department of Cardiology, University Hospital of Skåne, Lund/Malmö, Sweden; Wallenberg Centre for Molecular Medicine, Lund University, Lund, Sweden; Cardiovascular Research–Translational Studies, Clinical Sciences Malmö, Lund, Lund University, Sweden; Division of BioTherapeutics, Leiden Academic Centre for Drug Research, Leiden University, Leiden, The Netherlands; Department of Vascular Surgery, University Medical Center, Utrecht, The Netherlands; Division of BioTherapeutics, Leiden Academic Centre for Drug Research, Leiden University, Leiden, The Netherlands; Division of BioTherapeutics, Leiden Academic Centre for Drug Research, Leiden University, Leiden, The Netherlands; Department of Medical Biochemistry, Amsterdam UMC, University of Amsterdam, Meibergdreef 9, 1105 AZ, Amsterdam, The Netherlands; Amsterdam Cardiovascular Sciences (ACS), Atherosclerosis & Ischemic Syndromes, Amsterdam UMC, Meibergdreef 9, 1105 AZ, Amsterdam, The Netherlands; Amsterdam Institute for Immunology and Infectious Diseases (AII), Inflammatory Diseases, Amsterdam UMC, Meibergdreef 9, 1105 AZ, Amsterdam, The Netherlands; Department of Vascular Surgery, University Medical Center, Utrecht, The Netherlands; Department of Vascular Surgery, University Medical Center, Utrecht, The Netherlands; Amsterdam Cardiovascular Sciences (ACS), Atherosclerosis & Ischemic Syndromes, Amsterdam UMC, Meibergdreef 9, 1105 AZ, Amsterdam, The Netherlands; Amsterdam Institute for Immunology and Infectious Diseases (AII), Inflammatory Diseases, Amsterdam UMC, Meibergdreef 9, 1105 AZ, Amsterdam, The Netherlands; Department of Experimental Vascular Medicine, Amsterdam UMC, University of Amsterdam, Amsterdam, The Netherlands; Laboratory of Angiogenesis and Vascular Metabolism, Department of Oncology, KU Leuven, Leuven, Belgium; Laboratory of Angiogenesis and Vascular Metabolism, Center for Cancer Biology, VIB, Leuven, Belgium; Department of Surgery, Haaglanden Medisch Centrum Westeinde, The Hague, The Netherlands; Department of Surgery, Haaglanden Medisch Centrum Westeinde, The Hague, The Netherlands; Amsterdam Cardiovascular Sciences (ACS), Atherosclerosis & Ischemic Syndromes, Amsterdam UMC, Meibergdreef 9, 1105 AZ, Amsterdam, The Netherlands; Amsterdam Institute for Immunology and Infectious Diseases (AII), Inflammatory Diseases, Amsterdam UMC, Meibergdreef 9, 1105 AZ, Amsterdam, The Netherlands; Department of Experimental Vascular Medicine, Amsterdam UMC, University of Amsterdam, Amsterdam, The Netherlands; Department of Medical Biochemistry, Amsterdam UMC, University of Amsterdam, Meibergdreef 9, 1105 AZ, Amsterdam, The Netherlands; Amsterdam Cardiovascular Sciences (ACS), Atherosclerosis & Ischemic Syndromes, Amsterdam UMC, Meibergdreef 9, 1105 AZ, Amsterdam, The Netherlands; Amsterdam Institute for Immunology and Infectious Diseases (AII), Inflammatory Diseases, Amsterdam UMC, Meibergdreef 9, 1105 AZ, Amsterdam, The Netherlands; Department of Pathology, Maastricht University Medical Centre+, Maastricht University, Maastricht, The Netherlands; Cardiovascular Research Institute Maastricht (CARIM), Maastricht University, Maastricht, The Netherlands; Division of BioTherapeutics, Leiden Academic Centre for Drug Research, Leiden University, Leiden, The Netherlands; Cardiovascular Research–Translational Studies, Clinical Sciences Malmö, Lund, Lund University, Sweden; Department of Cardiology, University Hospital of Skåne, Lund/Malmö, Sweden; Laboratory of Clinical Chemistry and Haematology, University Medical Center, Heidelberglaan 100, 3584 CX, Utrecht, The Netherlands; Laboratory of Clinical Chemistry and Haematology, University Medical Center, Heidelberglaan 100, 3584 CX, Utrecht, The Netherlands; Experimental Cardiology, Department of Heart and Lungs, University Medical Centre Utrecht, Heidelberglaan 100, 3584 CX, Utrecht, The Netherlands; Department of Medical Biochemistry, Amsterdam UMC, University of Amsterdam, Meibergdreef 9, 1105 AZ, Amsterdam, The Netherlands; Amsterdam Cardiovascular Sciences (ACS), Atherosclerosis & Ischemic Syndromes, Amsterdam UMC, Meibergdreef 9, 1105 AZ, Amsterdam, The Netherlands; Amsterdam Institute for Immunology and Infectious Diseases (AII), Inflammatory Diseases, Amsterdam UMC, Meibergdreef 9, 1105 AZ, Amsterdam, The Netherlands

**Keywords:** Macrophages, CVD, atherosclerosis, biomarkers, TREM1, PLIN2, iLAM, inflammation

## Abstract

**Background and Aims:**

Atherosclerosis is a chronic lipid-driven inflammatory disease and one of the leading underlying causes of cardiovascular morbidity and mortality in Western society. Macrophages are key players in atherosclerotic development. Although the cellular composition of carotid atherosclerotic lesions has been determined, macrophage population definitions lack granularity and lineage data. Moreover, to date no direct link has been established between cellular content of atherosclerotic lesions and secondary clinical outcome. This study is aimed at characterization of atherosclerotic lesion macrophages and identification of plaque cell types and marker genes that predict the risk of secondary major adverse cardiovascular events in a clinical setting.

**Methods:**

Single-cell RNA sequencing on blood and plaques from 46 carotid endarterectomy patients enrolled in the AtheroExpress cohort. Deconvolution was done on bulk transcriptome data from 656 AtheroExpress patients, and findings were validated in 82 patients enrolled in the Carotid Plaque Imaging Project.

**Results:**

Four major archetypes of plaque macrophages were identified: inflammatory macrophages, lipid-associated macrophages (LAMs), tissue-resident-like LAMs, and inflammatory LAMs. Cellular trajectory and fate analyses revealed that these are derived from both classical and non-classical monocytes. Functionally, this study demonstrated the capacity of monocytes to differentiate into inflammatory LAMs via inflammatory- or resident-like LAM and LAM stages. Next, the AtheroExpress bulk RNA-seq cohort was deconvoluted. Macrophages were shown to be the only cell population significantly associated with both symptoms at time of surgery and increased risk of major adverse cardiovascular events during a 3-year follow-up period. Within the macrophage population, mostly LAM and inflammatory LAM foam cell markers such as PLIN2 and TREM1 were associated with an increased risk of major adverse cardiovascular events after 3-year follow-up. These associations were validated in the Carotid Plaque Imaging Project cohort.

**Conclusions:**

Together, these findings provide critical insights into the functional differences and origin of macrophage subpopulations in human atherosclerosis and show their clinical significance and risk prediction value in relation to future cardiovascular events.

## Structured Graphical Abstract

**Structured Graphical Abstract:**
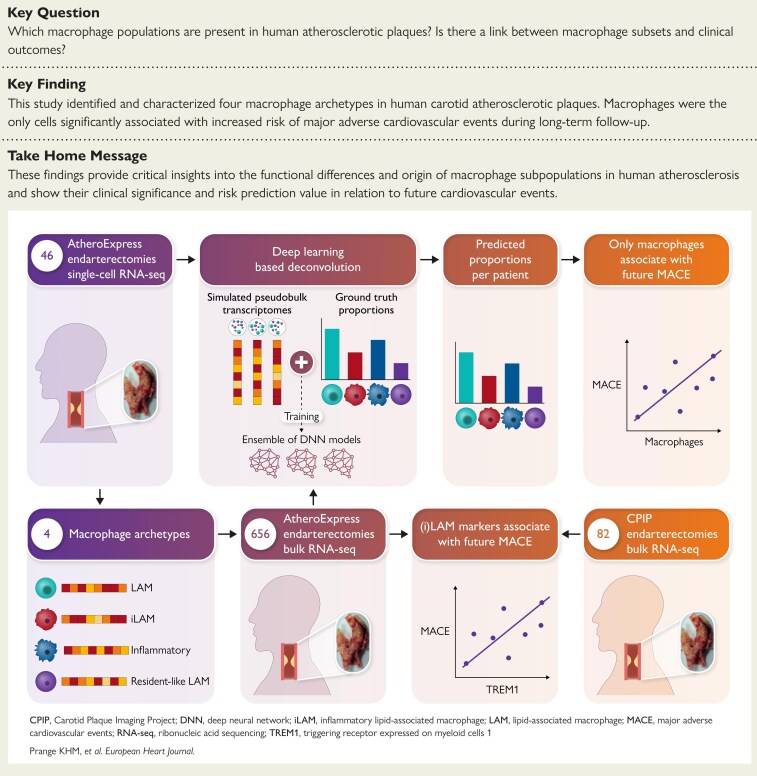

**See the editorial comment for this article ‘Lipid-associated macrophages in carotid plaques and cardiovascular outcomes: linking mechanisms and clinical management’, by L. Dib**  ***et al*****., https://doi.org/10.1093/eurheartj/ehag289.**

Translational perspective Macrophages are key players in atherosclerosis, but definitions of macrophage subsets in human plaques are limited, and no direct link has been established between cellular content of lesions and future clinical outcome. Using single-cell RNA sequencing on 46 carotid endarterectomy samples and deconvoluted bulk transcriptomes of two large atherosclerosis cohorts, this study identifies and characterizes four plaque macrophage archetypes and shows that macrophages are the only cells significantly associating with increased risk of major adverse cardiovascular events during long-term follow-up. These findings provide critical insights into macrophage subpopulations in atherosclerosis and show their importance for future cardiovascular events.

## Introduction

Atherosclerosis, the predominant cause of cardiovascular events,^1^ is driven by lipid accumulation in the subendothelial compartment, which induces a cellular immune response in the arterial wall. Macrophages are central players in this process aimed at clearing accumulated lipids and cellular debris giving rise to foam cells.^2–4^ Foam cell formation is considered a crucial step contributing to the low-grade inflammatory state, hallmarking progression of atherosclerotic lesions.^5,6^ Anti-inflammatory interventions have been shown to reduce the residual cardiovascular risk in patients on top of optimal lipid-lowering therapies.^7,8^ Interleukin (IL)-1β inhibition and other anti-inflammatory treatments were found to reduce major adverse cardiovascular event (MACE) rates by 15%–25%, independent of any change in lipid parameters.^9–11^ Conversely, aggressive lipid-lowering therapies have been reported to attenuate the inflammatory status in atherosclerotic plaques, highlighting the close interplay between lipids and inflammation.^8,12^ Since lesional macrophages have both inflammatory and lipid scavenging functions, this suggests that both processes are embroiled in atherogenesis. In support of the complex role of macrophages, a wide variety of macrophage subtypes are present in the atherosclerotic plaque, filling different niches of the pathophysiological process.^13–15^ Intriguingly, despite overwhelming data suggesting a role for macrophages and macrophage-associated inflammatory processes in human atherosclerotic disease, direct evidence linking plaque macrophage content and adverse clinical outcome in patients is completely lacking.

Previously, we and others identified distinct inflammatory and foamy macrophage subsets within human atherosclerotic plaques by single-cell RNA sequencing (scRNA-seq).^15–18^ Moreover, it was shown that a population of *TREM1*^hi^ inflammatory foam cells known as inflammatory lipid-associated macrophages (iLAMs), which evolve from *TREM2*^hi^ lipid-associated macrophages (LAMs), associate with symptomatic carotid disease.^15^ Here, we set out to investigate the molecular characteristics of (i)LAM differentiation, the relation of human plaque macrophages to peripheral blood monocytes, and the association of plaque cell types in general and macrophage subtypes specifically to clinical characteristics of patients.

For this, we expanded our cohort^16^ to 46 carotid endarterectomy patients and added paired peripheral blood mononuclear cell (PBMC) samples from a subset of patients. The expanded cohort was used to define functionally and molecularly distinct plaque macrophage types, and we were able to compare blood monocytes to plaque macrophages and determine cellular origins and fates within the plaque. Subsequently, we validated these trajectories and characteristics *in vitro* in primary human monocyte to macrophage differentiation. Lastly, we used a deep learning–based deconvolution approach to quantify cellular subsets in lesions from a large cohort of 656 endarterectomy patients and link plaque cell type composition to the clinical traits of patients at time of surgery and after a 3-year follow-up period. Interestingly, we found that macrophages were associated with cardiovascular symptoms at time of surgery and predicted the risk of MACE during 3-year follow up. Moreover, specifically expression of iLAM and LAM marker genes was associated with MACE in 3-year follow-up and improved the clinical risk factor-based prediction models for secondary MACE, altogether strengthening the case for, e.g. TREM1 levels as a predictor for symptomatic carotid atherosclerosis development. This was confirmed in a smaller independent cohort (*Structured Graphical Abstract*).

## Methods

See supplemental methods.

## Results

### Human plaque macrophages cluster into four major archetypes

**Figure 1:**
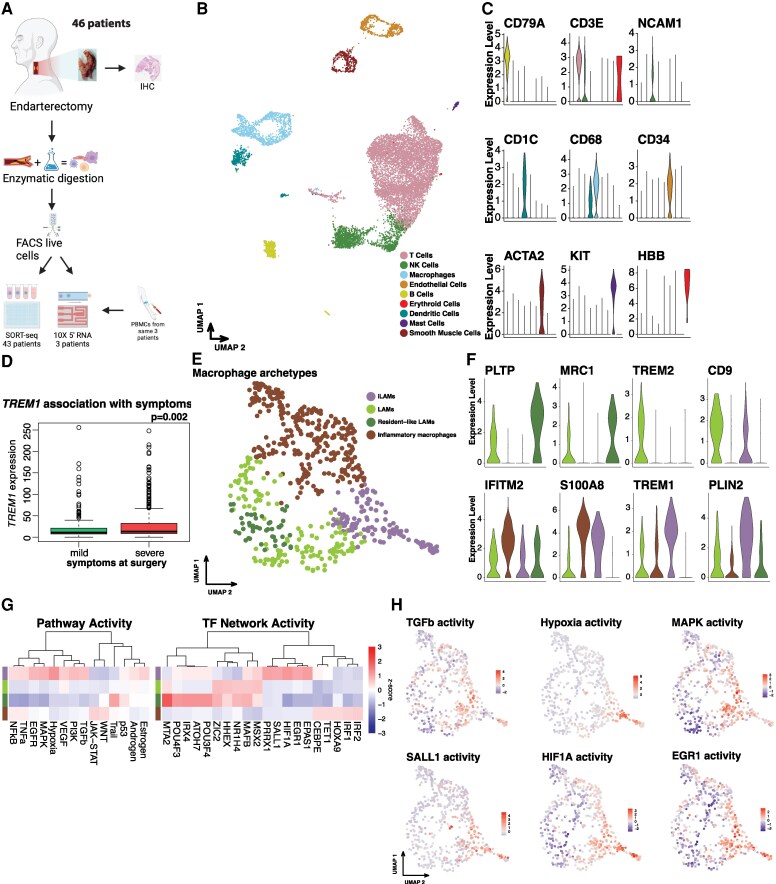
Plaque macrophages can be divided into four archetypes. (*A*) Schematic overview of experimental procedures. (*B*) UMAP of cell populations present in 46 human carotid plaques. (*C*) Violin plots of cell type-defining marker genes per population. (*D*) Violin plot showing *TREM1* expression correlation over patients with mild (asymptomatic + ocular) vs severe (transient ischaemic attack + stroke) symptoms at surgery. Expression data from 656 patients bulk RNA sequencing (Counts Per Million (CPM)). Statistical difference was obtained by Wilcoxon test. (*E*) UMAP of the subset of plaque macrophages. (*F*) Violin plots of cell type-defining marker genes per macrophage population. (*G*) Heat map of pathway (left) and transcription factor network activity in the macrophage populations. Scale: z-score of activity. (*H*) UMAPs of plaque macrophages showing pathway or transcription factor-network activity. Scale: z-score of activity

To gain increased insights and allow linkage of plaque cell composition to clinically relevant traits, we extended our previously published cohort of scRNA-seq data of carotid endarterectomy plaque specimen^16^ to 46 patients. Plaques from 43 patients were sequenced by a single-cell sorting-based method (SORT-seq). In addition, we generated libraries for three more patients using a microfluidics-based approach (10× genomics 5′ gene expression) (*Figure 1A*). This dual approach allowed to expand the breadth of our original cohort to better cover human plaque heterogeneity.

Libraries were analysed and integrated with correction for batch effects (see Supplementary data online, *Figure S1A*–C) as described in the Methods. This yielded a total of 11 779 cellular transcriptomes from human plaques. Cell types identified based on marker gene expression were B cells, dendritic cells, endothelial cells, erythroid cells, macrophages, mast cells, natural killer cells, smooth muscle cells, and T cells (*Figure 1B* and *C*; Supplementary data online, *Figure S1D*). Cell type abundances were in line with previous work,^15,16^ with T cells being the most abundant (∼60%) and macrophages accounting for 10% of all cells present in human carotid plaques (see Supplementary data online, *Figure S1E*).

Recently, *TREM1^hi^* macrophage foam cells were shown to associate with symptomatic carotid disease.^15^ By interrogating our bulk RNA-seq data of 656 patients from the AtheroExpress (AE) cohort, we could confirm that *TREM1* expression is higher in patients with severe symptoms [i.e. stroke or transient ischaemic attack (TIA)] prior to surgery (*Figure 1D*). In line, we could show in our scRNA-seq data that *TREM1* expression is higher in macrophages from atheromatous (fat > 40%) plaques (see Supplementary data online, *Figure S1F*) compared with fibroatheromatous or fibrous plaques.

Subsequently, we focused our analysis of scRNA-seq data on macrophages. We identified four main macrophage archetypes, which we classified as inflammatory macrophages, LAMs, vascular-wall-resident-macrophage-like (resident-like) LAMs, and iLAMs (*Figure 1E*), based on analysis of top 15 marker genes per population (see Supplementary data online, *Figure S1G*) as well as relevant canonical markers for these macrophage phenotypes (*Figure 1F*). The resident-like LAMs to a great extent share marker gene expression with the LAMs but were negative for *TREM2* and higher in the expression of residence markers such as *MRC1* (*Figure 1F*; Supplementary data online, *Figure S1H*). All populations consisted of cells sourced from both library preparation methods (see Supplementary data online, *Figure S1I*) and a plurality of patients (see Supplementary data online, *Figure S1J* and *K*), demonstrating no marked per method or per patient influence on population distribution.

Next, we investigated the molecular mechanisms distinguishing the four macrophage populations. For this, we calculated biological pathway and transcription factor (TF) network activities in the four subsets. Inflammatory lipid-associated macrophages were notably driven by the hypoxia pathway and associated TF networks for HIF1A and HIF1B (EPAS1) (see Supplementary data online, *Figure S1G* and *H*). Next to that, iLAMs were driven by, e.g. Early Growth Response 1 (EGR1) and Spalt Like Transcription Factor 1 (SALL1) TFs and mitogen-activated protein kinase (MAPK) and Transforming Growth Factor Beta (TGFβ signalling pathways. Inflammatory lipid-associated macrophages and inflammatory macrophages were both enriched for Nuclear Factor Kappa B (NFκΒ) and Tumor Necrosis Factor (TNF) pathway activity, while inflammatory macrophages alone were enriched for, e.g. Interferon Regulatory Factor 1 (IRF1) and Interferon Regulatory Factor 2 (IRF2) TFs and WNT (named after the Wingless/Int-1 gene family) and Homeobox A9 (HOXA9) signalling. Interestingly, inflammatory macrophages were also enriched for Janus Kinase - Signal Transducer and Activator of Transcription (JAK-STAT) activity, required for inflammatory activation of macrophages of monocytic origin,^19^ while the LAMs and resident-like LAMs show reciprocal anti-inflammatory V-maf musculoaponeurotic fibrosarcoma oncogene homolog B (MAFB) activity.^20^ In line, LAMs and resident-like LAMs show enrichment of factors related to cell growth and haematopoietic development, such as HHEX,^21^ MSX2,^22^ and MAFB.^23,24^ However, resident-like LAMs alone were enriched for factors such as MTA2 and ZIC2, which have been linked to an M2-like anti-inflammatory phenotype^25,26^ (see Supplementary data online, *Figure S1L*). The demarcated TF-network and pathway activity of the resident-like LAM population together with its unique expression pattern of canonical resident macrophage markers, e.g. FOLR2, emphasizes that this population is different from the general LAM population, even though both appear close together in Uniform Manifold Approximation and Projection (UMAP) space.

### Integration with external datasets confirms macrophage subpopulations

To validate the robustness of our macrophage clusters, we integrated our dataset with four independent scRNA-seq studies on human carotid plaques. Across these, all major macrophage archetypes were supported by at least one external dataset. Of note, the Pan *et al*.^27^ and Wirka *et al*.^28^ datasets did not contain iLAM foam cells and captured fewer inflammatory macrophages, likely reflecting the impact of dissociation and library preparation strategies (see Supplementary data online, *Figure S2A*). In contrast, the Dib *et al*.^15^ and Horstmann *et al*.^18^ studies provided a more balanced representation of all macrophage populations (see Supplementary data online, *Figure S2B*). Integration of these two datasets with our own resulted in a combined set of 6983 macrophages. Mapping our archetype labels onto this integrated dataset showed clean separation of clusters, confirming that the original archetypes remain robust when extended to independent studies (see Supplementary data online, *Figure S2C*). The only partial overlap was observed between the resident-like LAM and the LAM populations. Examination of canonical markers (see Supplementary data online, *Figure S2D*) showed that both clusters share markers such as CD163, MRC1, FOLR2, and C1QB, consistent with their proximity in UMAP space. However, the resident-like LAM cluster was distinguished by a complete absence of TREM2 expression, a hallmark LAM marker, and by exclusive expression of CCL18, typically associated with tumour-associated macrophages.^29,30^ This suggests that the resident-like LAM cluster represents a functionally divergent LAM subtype. Clustering the integrated dataset (30 PCA dimensions, resolution .5) and mapping the resident-like LAM cells as described above delineated 10 distinct macrophage phenotypes (see Supplementary data online, *Figure S2E*). These phenotypes were consistently identified across most patients in all three datasets (see Supplementary data online, *Figure S2F* and *G*), with the exception of the more fragile stressed iLAM subtypes (C9 and C10), observed in ∼25% of patients. Each population displayed distinct marker genes and pathway enrichments (see Supplementary data online, *Figure S2H* and *I*). Inflammatory lipid-associated macrophage populations, in particular, were enriched for pathways related to proliferation, including Myc targets^31,32^ and MTORC1 signalling,^33^ although expression of the canonical proliferation marker Ki-67 was sparse, low, and restricted to the C5 LAM population (see Supplementary data online, *Figure S2J* and *K*). Taken together, the convergence of pathways and marker expression within archetypal boundaries indicates that these 10 phenotypes represent nuanced heterogeneity within the broader framework of four macrophage archetypes.

### Macrophage archetype presence in human carotid atherosclerotic plaques could be confirmed by fluorescence-activated cell sorting for key marker proteins

To validate the macrophage archetype markers identified by transcriptomics, we next examined their protein expression using immunohistochemistry and spectral flow cytometry. Immunostaining of human lesions with CD14 (myeloid cells), S100A9 (inflammatory macrophages), CD9 (LAMs), CD206 (resident-like LAMs), and PLIN2 (iLAMs) confirmed the presence of these markers at the protein level^29,34,35^ (see Supplementary data online, *Figure S3A*, *Supplemental resource table*). Building on this, we designed an antibody panel including S100A9, FOLR2, CD9, CD68, CD14, CD163, CD11b, TREM2, TREM1, CD206 (MRC1), and CD204 (MSR1) and applied it to digested cells from three human lesions. Clustering of the gated macrophages revealed five distinct populations (see Supplementary data online, *Figure S3B* and *C*). These were defined by broad CD11b and CD14 positivity and a gradient of CD163 and CD68 expression (see Supplementary data online, *Figure S3D*). Clusters 1 and 4 showed characteristics of both LAMs (TREM2) and resident-like LAMs (CD206, FOLR2), consistent with the close relationship of these populations. Clusters 3 and 5 were enriched for the inflammatory macrophage marker S100A9, while the small Cluster 2 expressed the iLAM marker TREM1 alongside TREM2, with other markers largely absent. Among the tested markers, CD204 did not resolve clear macrophage subsets, and CD9 labelled multiple populations, displaying less specificity than observed in scRNA-seq. Overall, protein-level validation confirmed the presence of macrophage subsets corresponding to scRNA-seq archetypes while also illustrating the expected differences between transcriptomic and protein readouts.

### Both classical and non-classical blood monocytes contribute to the pool of inflammatory plaque macrophages

Marker genes of the macrophage archetypes share conspicuous similarities and differences. The LAM and resident-like LAM populations share a common transcriptional programme, and most cells express monocyte markers such as *SELL* and *FCN1* (see Supplementary data online, *Figure S2H* and *I*) while lacking expression of proliferation genes (see Supplementary data online, *Figure S2J* and *K*) and gene programmes for local proliferation of tissue-resident cells (see Supplementary data online, *Figure S2I*). Therefore, we next investigated the possible differentiation paths of plaque macrophages. For this, we first analysed the connection between plaque macrophage populations and monocyte populations. Paired PBMC scRNA-seq libraries were created from the same three patients as the plaque libraries on the microfluidics platform (*Figure 1A*) and integrated with the plaque libraries as described above.

**Figure 2:**
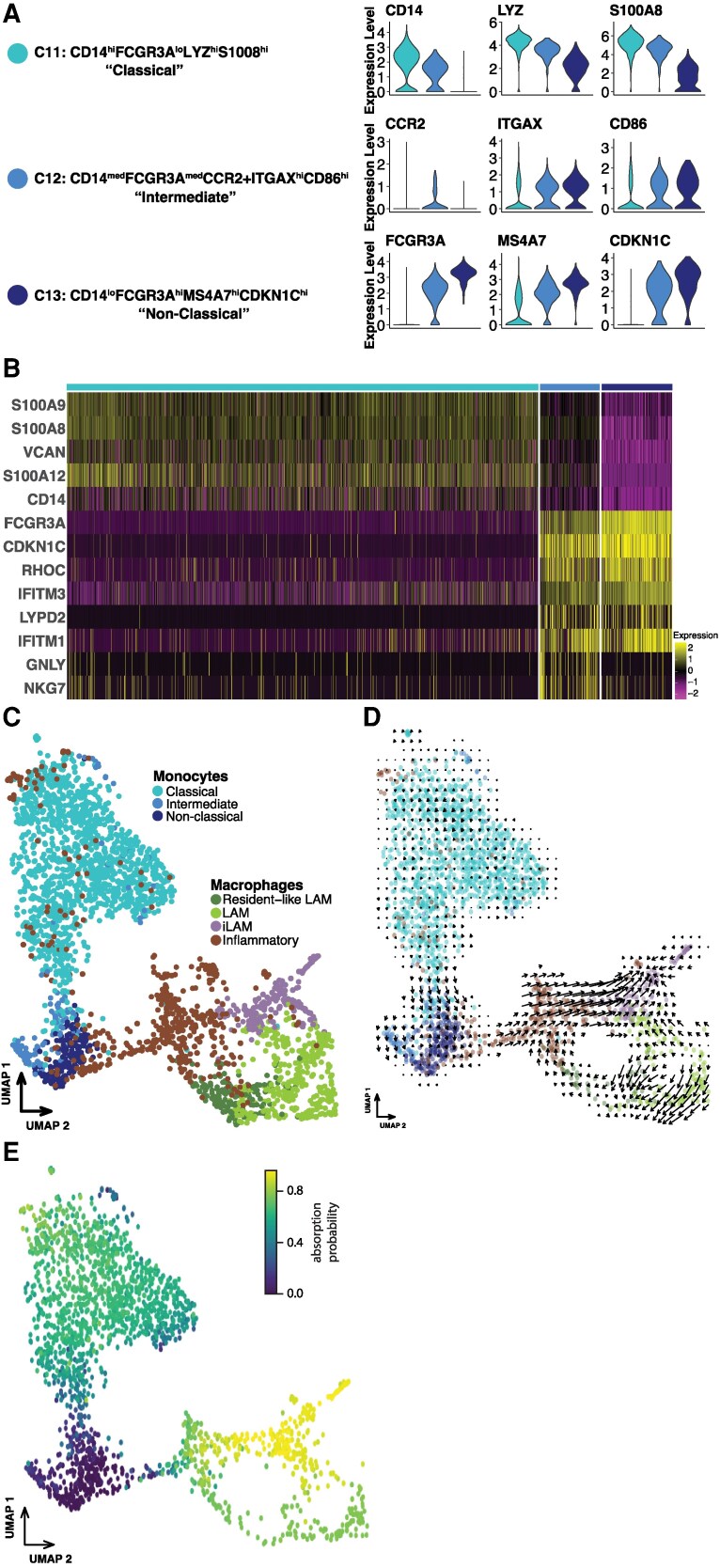
Plaque macrophages converge into inflammatory lipid-associated macrophages as terminal cell type. (*A*) Violin plots of type-defining marker gene expression per monocyte population. (*B*) Heat map showing the top five marker genes per monocyte population. (*C*) Integrated UMAP of plaque macrophage and peripheral blood mononuclear cell monocyte populations. (*D*) UMAP of all macrophage and monocyte populations overlayed with cellular trajectories as calculated by Velocyto (arrows). (*E*) UMAP of all macrophage and monocyte populations showing inflammatory lipid-associated macrophage lineage commitment per cell as defined by CellRank. Scale: absorption probability

Monocytes were separated from the PBMCs, yielding 1715 monocytes (see Supplementary data online, *Figure S4A*). These could be divided into three populations: classical (C11), intermediate (C12), and non-classical monocytes (C13), based on marker gene expression^30^ (*Figure 2A* and *B*). Monocytes were embedded with the macrophages by UMAP. The inflammatory macrophage plaque population and the classical and non-classical monocyte PBMC populations partially overlapped in UMAP space (*Figure 2C*). This phenomenon, together with the many monocytic marker genes such as *FCG3A*, *SELL*, *FCN1*, and *CCR2* being expressed in the plaque macrophages, suggests a close connection between the peripheral blood monocytes and plaque macrophages and prompted us to investigate the interconnectedness of these populations.

We first analysed cell trajectories with monocle 3,^36^ which connected circulating monocytes to plaque macrophages, primarily linking non-classical monocytes to inflammatory macrophages (see Supplementary data online, *Figure S4B*). To infer directionality, we performed RNA velocity on monocytes and macrophages from the 10× libraries^37^ (*Figure 2D*). This confirmed a transition from (non-)classical monocytes → inflammatory macrophages → iLAMs. Notably, RNA velocity also revealed a second origin at the resident-like LAM/LAM interface, suggesting both monocyte-derived and resident-like cells contribute to iLAMs, supporting the TREM2^hi^ LAM → TREM1^hi^ iLAM transition.^15^ Separate RNA velocity analysis per patient showed similar results despite density differences (see Supplementary data online, *Figure S4C*). Pseudotime analysis further confirmed classical monocytes (C10) as transcriptionally most distinct from LAM populations (see Supplementary data online, *Figure S3D*), while monocytes and inflammatory macrophages showed little pseudotime variation, reinforcing the inferred trajectory. Finally, combining trajectory, pseudotime, and RNA velocity using CellRank^38^ revealed two main lineages: (i) PBMC monocytes differentiating classical → non-classical (see Supplementary data online, *Figure S4E*) and (ii) classical monocytes → inflammatory macrophages → (resident-like) LAMs → iLAMs (*Figure 2E*).

Collectively, our data reveal two main axes from blood monocytes to plaque macrophages: (i) circulating monocytes → inflammatory macrophages → iLAMs, driven by chemotaxis, proliferation, inflammation, hypoxia, and lipid uptake, and (ii) inflammatory macrophages → (resident-like) LAMs → iLAMs, linked to lipid uptake, alternative activation, inflammation, and metabolism (*Figure 1G* and *H*; Supplementary data online, *Figure S1K* and *I*). The triggers for transitions through the TREM2⁻ resident-like LAM state remain unclear. In both axes, iLAMs represent the terminal population, marked by hypoxia, stress, apoptosis, and necrosis pathways.

### Macrophage differentiation axes can be recapitulated *in vitro*

**Figure 3:**
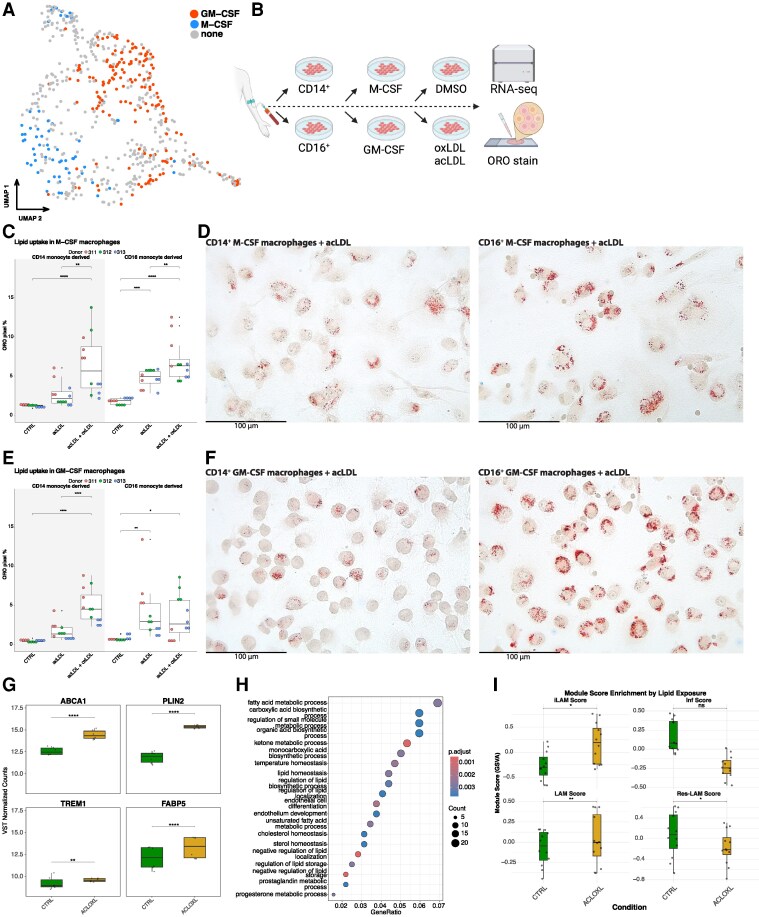
Macrophage differentiation axes can be recapitulated *in vitro*. (*A*) UMAP of plaque macrophages showing significant enrichment of macrophage colony-stimulating factor (blue) or granulocyte-macrophage colony-stimulating factor gene expression signatures as calculated by AUCell. (*B*) Experimental setup schematic: Monocyte to macrophage differentiation and response to lipids. (*C*) Boxplot showing the proportion of pixels coloured by oil-red-O in macrophage colony-stimulating factor. (*D*) Representative images of oil-red-O staining on CD14+ monocyte-derived (left) and CD16+ monocyte-derived (right) macrophage colony-stimulating factor. (*E*) Boxplot showing the proportion of pixels coloured by oil-red-o in granulocyte-macrophage colony-stimulating factor. (*F*) Representative images of oil-red-o staining on CD14+ monocyte-derived (left) and CD16+ monocyte-derived (right) granulocyte-macrophage colony-stimulating factor. (*G*) RNA-sequencing data of selected inflammatory lipid-associated macrophage marker gene expression in lipid-laden macrophages vs dimethylsulfoxide control. *H*) Top 20 enriched Gene Ontology (GO) terms in lipid laden macrophages vs DMSO control. (*I*) Enrichment of archetype modules derived from the single-cell RNA-sequencing data in lipid-laden macrophages vs dimethylsulfoxide control. DMSO, dimethylsulfoxide; M-CSF, macrophage colony-stimulating factor; GM-CSF, granulocyte-macrophage colony-stimulating factor; acLDL, acetylated LDL; oxLDL, oxidized LDL. Statistical differences were tested by one-way ANOVA with Tukey’s Honestly Significant Difference (HSD) *post hoc* test for pairwise comparisons. ns = not significant; *Padj < .05; **Padj < .01; ***Padj < . 001; ****Padj < .0001

Next, to investigate the capacity of both classical and non-classical monocytes to differentiate into iLAMs via either differentiation axis, we performed *in vitro* experiments. We compared two major differentiation cytokines commonly used for *in vitro* generation of macrophages,^31^ i.e. macrophage colony-stimulating factor (M-CSF) and granulocyte-macrophage colony-stimulating factor (GM-CSF). Enrichment analysis of M-CSF- and GM-CSF-stimulated macrophage gene expression signatures in our macrophage populations (*Figure 3A*) showed that the resident-like LAMs were dominated by M-CSF signatures, while inflammatory macrophages were more driven by GM-CSF.

We differentiated CD14^+^ (classical) and CD16^+^ (non-classical) monocytes with (G)M-CSF for 1 week, to model resident-like LAM and inflammatory plaque macrophage differentiation and subsequently stimulated cells with acetylated LDLs (acLDLs) and/or oxidized LDLs (oxLDLs) for 24 h as proxies to simulate macrophage exposure to various lipids within the plaque (*Figure 3B*). Transcriptomics analyses showed that GM-CSF-derived macrophages are indeed more inflammatory than their M-CSF counterparts (see Supplementary data online, *Figure S5A*). The M-CSF-derived macrophages conversely were enriched for replication related pathways and showed higher expression of resident-like marker genes such as FOLR2 and MMP9 (see Supplementary data online, *Figure S5B*). Oil-red-O (ORO) lipid staining revealed that both M-CSF- and GM-CSF-derived macrophages have the capacity to take up lipids and become foam cells (*Figure 3C–F*). Interestingly, while both CD14^+^ (classical) and CD16^+^ (non-classical) monocyte-derived macrophages can scavenge lipids, CD16^+^-derived macrophages showed a higher capacity to take up acLDL specifically (*Figure 3C–F*; Supplementary data online, *Figure S5C* and *D*). This difference disappeared after addition of oxLDL, which suggests macrophages from different sources might take on a different phenotype in the plaque dependent on the lipid species available in the lesion environment. Transcriptomic analysis further revealed induction of an iLAM-like state in both M-CSF- and GM-CSF-derived macrophages upon lipid exposure, with increased expression of markers such as ABCA1, PLIN2, TREM1, and FABP5 identified in our single-cell data (*Figures 1F* and *3G*; Supplementary data online, *Figure S1G* and *Data S1*). Comparison of lipid exposed macrophages to dimethylsulfoxide (DMSO) controls yielded 924 differentially expressed genes (*P*adj < .05, |log₂FC| > 1), including iLAM markers PLIN2 and TREM1 (see Supplementary data online, *Figure S5E* and *F* and *Data S2*). Enrichment analysis highlighted lipid and cholesterol metabolism pathways (*Figure 3H*), consistent with an iLAM phenotype. Finally, archetype-module scoring confirmed that lipid exposure induced signatures of both iLAMs and LAMs, whereas resident-like LAM signatures decreased (*Figure 3I*).

Together, these data demonstrate that, indeed, both classical and non-classical monocytes can differentiate into macrophages with an iLAM-like phenotype after exposure to lipids. This holds true for resident-like LAMs modelled by M-CSF and inflammatory macrophages modelled by GM-CSF.

### Plaque macrophage content is associated with perioperative symptoms and future risk of major adverse cardiovascular events

**Figure 4:**
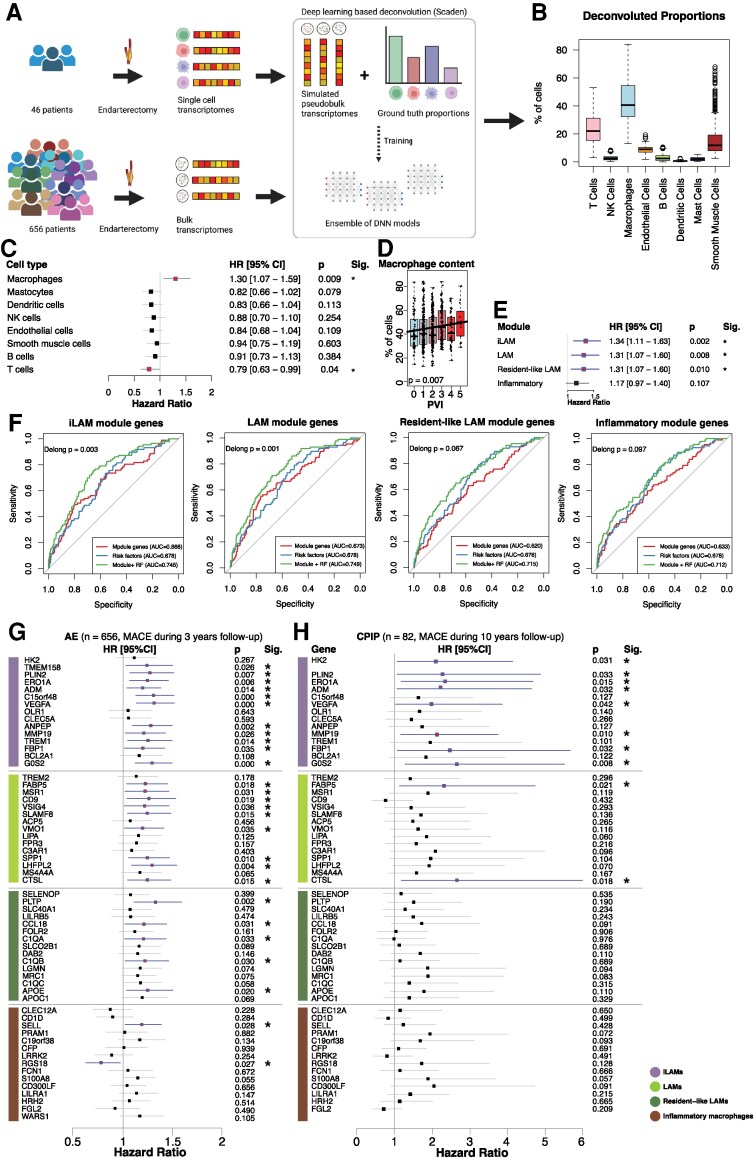
Human plaque macrophage content associates with major adverse cardiovascular event. (*A*) Schematic description of cell type deconvolution of bulk RNA-sequencing data using the Scaden package. (*B*) Violin plot showing deconvoluted cell type proportions. (*C*) Forest plot showing the hazard ratio for the association of major adverse cardiovascular event with various cell types at 3-year follow-up. Red boxes and blue lines denote a significant association (*P* < .05). (*D*) Violin plot showing the correlation between the number of CD68-positive cells (macrophages) and six bins of plaque vulnerability index (PVI). (*E*) Forest plot showing the hazard ratio for the association of major adverse cardiovascular event with the archetype modules at 3-year follow-up. Red boxes and blue lines denote a significant association (*P* < .05). (*F*) Receiver Operating Characteristic (ROC) curves showing the added value of the archetype genes over known risk factors (age, sex, body mass index, HDL cholesterol, glomerular filtration rate, peripheral arterial occlusive disease, diabetes and history of coronary artery disease) in risk prediction model. (*G*) Forest plot showing association of individual marker gene expression values and major adverse cardiovascular event after 3-year follow-up in the AtheroExpress cohort. (*H*) Forest plot showing association of individual marker gene expression values and MACE up to 10 years of follow-up in the Carotid Plaque Imaging Project cohort. All associations with major adverse cardiovascular event are corrected for age, sex, body mass index, HDL cholesterol, glomerular filtration rate, peripheral arterial occlusive disease, diabetes, and history of coronary artery disease). AE, AtheroExpress; CPIP, Carotid Plaque Imaging Project; HR, hazard ratio; CI, confidence interval

To test the connection between cellular subsets of atherosclerotic plaques and clinical traits, we used our scRNA-seq dataset to deconvolute our bulk RNA-seq data of 656 patients from the AE cohort (*Table 1*). Briefly, we simulated pseudobulk training datasets using our single-cell transcriptomes, with which we trained an ensemble of deep neural network models, to deconvolute cell type fractions^32^ (*Figure 4A* and *B*). The resulting values of the macrophage fractions showed a consistent correlation across various deconvolution methods (see Supplementary data online, *Figure S6A*) and were positively correlated with the number of CD68-positive cells observed by immunohistological examination (*Table 2*).^32,39–42^ Interestingly, investigating cell populations in the 656 patients revealed that the presence of cerebrovascular events (stroke or TIA) prior to carotid endarterectomy was positively associated with plaque macrophage content (see Supplementary data online, *Figure S6B*) compared with patients who were subjected to elective carotid endarterectomy (general risk factors and severe stenosis > 70%) or had isolated temporary ocular blindness [beta = .27, 95% confidence interval (CI) .10–.44, *P* = .0024]. Other cell types did not associate (T and NK cells, smooth muscle cells, mast cells), or negatively (B cells, endothelial cells, dendritic cells) associated with a cerebrovascular event.

**Table 1:** AtheroExpress patient baseline characteristics

	Overall	No MACE within 3 years	MACE within 3 years	*P*-value
*n*	649	561	88	
Age (years), mean (SD)	68.55 (8.86)	68.17 (8.86)	70.99 (8.49)	.005
Male sex, *n* (%)	482 (74.3)	410 (73.1)	72 (81.8)	.107
BMI (kg/m^2^) mean (SD)	26.61 (3.77)	26.51 (3.72)	27.27 (4.07)	.094
Current smoker, *n* (%)	230 (36.0)	195 (35.2)	35 (41.2)	.343
eGFR (mL/min/1.73 m^2^), mean (SD)	72.83 (20.92)	73.81 (20.60)	66.56 (21.94)	.003
Total cholesterol (mmol/L), mean (SD)	4.67 (1.25)	4.71 (1.26)	4.45 (1.13)	.121
LDL cholesterol (mmol/L), mean (SD)	2.78 (1.04)	2.81 (1.04)	2.65 (1.01)	.291
HDL cholesterol (mmol/L), mean (SD)	1.14 (.38)	1.16 (.37)	1.06 (.40)	.054
Triglycerides (mmol/L), mean (SD)	1.63 (.94)	1.61 (.91)	1.71 (1.08)	.451
Diabetes mellitus, *n* (%)	140 (21.6)	110 (19.6)	30 (34.1)	.003
Hypertension, *n* (%)	457 (72.5)	389 (71.4)	68 (80.0)	.127
CAD history, *n* (%)	430 (66.4)	379 (67.6)	51 (58.6)	.129
Stroke history, *n* (%)	450 (69.3)	394 (70.2)	56 (63.6)	.261
Peripheral arterial occlusive disease, *n* (%)	143 (22.1)	115 (20.5)	28 (32.2)	.021
Symptoms at inclusion, *n* (%)				.872
Asymptomatic	97 (15.2)	84 (15.3)	13 (14.8)	
Ocular	108 (17.0)	95 (17.3)	13 (14.8)	
Stroke	161 (25.3)	136 (24.8)	25 (28.4)	
TIA	271 (42.5)	234 (42.6)	37 (42.0)	
Statin treatment, *n* (%)	483 (74.5)	422 (75.4)	61 (69.3)	.281
Antiplatelet treatment, *n* (%)	580 (89.5)	504 (90.0)	76 (86.4)	.397
Plaque phenotype, *n* (%)				.773
Atheromatous	188 (29.4)	165 (29.8)	23 (26.4)	
Fibroatheromatous	241 (37.7)	208 (37.6)	33 (37.9)	
Fibrous	211 (33.0)	180 (32.5)	31 (35.6)	

BMI, body mass index; CAD, coronary artery disease; eGFR, estimated glomerular filtration rate; MACE, major adverse cardiovascular events; SD, standard deviation; TIA, transient ischaemic attack

**Table 2:** Deconvoluted proportions compared with histology

Cell population	Histology score	Bisque^39^	CIBERSORTx^40^	MuSiC^41^	NNLS^42^	Scaden^32^
Macrophages	CD68	.094***	.096***	.089***	.09***	.1***
	Fat	.132***	.132***	.113***	.105***	.114***
	ACTA2: CD68	−.115***	−.134***	−.096***	−.088***	−.1***
	Plaque phenotype	−.123***	−.127***	−.1***	−.088***	−.1***

Associations between deconvolved proportions and related histological features were computed with Kendall rank correlation. Plaque phenotypes were converted to scores from 1 to 3, from atheromatous to fibrous.

***P < .01.

Subsequently, we linked our cellular subset quantifications to the risk of a MACE, during a 3-year follow-up after surgery. Excitingly, here plaque macrophage content was associated with a higher risk for MACE [Cox regression, hazard ratio (HR) 1.30, 95% CI 1.07–1.59, *P* = .009, adjusted for sex, age, body mass index, HDL cholesterol, kidney function, peripheral arterial occlusive disease, diabetes, and history of coronary artery disease], while none of the other cell population’s presence increased the risk of MACE (*Figure 4C*). In line, deconvoluted plaque macrophage content was positively associated with histologically determined plaque vulnerability index of lesions from 638 patients taken from the AE cohort (*Figure 4D*). Moreover, we validated our approach by deconvoluting the bulk AE cohort using the independent Tabula Sapiens (TS) human single-cell transcriptomics reference dataset^43^ instead of our own scRNA-seq data. In this way, we removed possible bias introduced by using single-cell transcriptomics data from the same cohort as the bulk dataset for the deconvolution analysis. These analyses confirmed the correlation of MACE during a 3-year-follow-up (see Supplementary data online, *Figure S6C*) and stroke/TIA prior to surgery (see Supplementary data online, *Figure S6D*) with monocyte (macrophage) content. Together, this shows that macrophages are critical for the pathophysiological mechanisms at play and clinical outcome of patients.

### Inflammatory lipid-associated macrophage marker gene expression associates with future major adverse cardiovascular event

Next, we wanted to assess the association of our four macrophage archetypes with clinical outcome. As deconvolution approaches tend to underperform on highly similar cell types such as macrophage subsets,^44^ we directly tested the associations of unique marker genes specifically expressed in plaque macrophages at the archetype level. For this, we first evaluated which of our previously empirically defined archetype markers (Supplementary data) are uniquely expressed in macrophages by performing k-means clustering on pseudobulk expression data aggregated by cell type (see Supplementary data online, *Figure S5E*). This yielded macrophage-specific marker gene clusters from which we used the top 15 by *P*-value to construct archetype gene modules. All three LAM modules were associated with MACE after 3-year follow-up, with the iLAMs being most significant. The inflammatory macrophage module did not significantly associate. Relative to a base model with classical risk factors [area under the curve (AUC) = .678], adding the expression of module genes for LAM and iLAM improved discrimination to AUC = .749 (ΔAUC = .071; *P*₍_DeLong_₎ = .001) and AUC = .745 (ΔAUC = .067; *P*₍_DeLong_₎ = .003), respectively. Adding top genes from resident and inflammatory macrophage signatures produced smaller gains, AUC = .715 (ΔAUC = .037; *P*₍_DeLong_₎ = .067) and AUC = .712 (ΔAUC = .034 *P*₍_DeLong_₎ = .097), which did not reach statistical significance (*Figure 4F*).

We next associated the expression of these cell type-specific archetype-module genes with the risk of MACE after 3-year follow-up in the AE bulk RNA-seq cohort of 656 patients (*Figure 4G*). Interestingly, the expression levels of 11 out of 15 iLAM and 9 out of 15 LAM and 5 out of 15 resident markers significantly increased with the risk of MACE, while only 1 of the 15 top marker genes for inflammatory macrophages showed positive association. Specifically, canonical iLAM markers *TREM1* (HR 1.26, 95% CI 1.05–1.50, *P* = .014) and *PLIN2* (HR 1.28, 95% CI 1.07–1.52, *P* = .007) associate with future MACE. While previous results link *TREM1* expression at surgery to symptomatic carotid disease^15^ (*Figure 1D*; Supplementary data online, *Figure S6F*), we here show the importance of *TREM1* and *PLIN2* expression for predicting the risk of future events up to 3 years after surgery. To validate our findings in an independent cohort, we finally investigated the association of the same marker genes with MACE up to 10 years of follow-up in 82 endarterectomy patients enrolled in the Carotid Plaque Imaging Project (CPIP) cohort^45,46^ (*Figure 4H*). Here, almost all markers followed the same trend of association as in our cohort, while iLAM markers such as *PLIN2* were significantly associated with MACE at follow-up. Together, we here show that macrophage content and specifically expression of subset markers are predictive for future events in endarterectomy patients.

## Discussion

In the present study, we analysed a cohort of 46 endarterectomy patients by scRNA-seq. This enabled us to identify plaque macrophage subpopulations and define their characteristics. Most interestingly, it allowed us to deconvolute data from 656 endarterectomy patients from the AE cohort. This revealed that macrophage content is not only uniquely associated with symptomatic disease (stroke or TIA) prior to carotid endarterectomy but that it also predicts the risk of recurrent events in these patients during a 3-year follow-up (see Supplementary data online, *Figure S6B*; *Figure 4C* and *D*). Our conclusion thus reinforces and directly validates the concept that macrophages are eminent players in the pathophysiology of human atherosclerosis^5^ and suggests that macrophage content at the time of surgery might be used as a parameter to identify patients at the highest risk of recurrent MACE.

Despite being regarded as ‘textbook knowledge’, direct evidence for the macrophage’s association for secondary risk in humans is surprisingly scarce. Previously our group published two studies that hinted at such a link, and both relied on indirect measurements: one used the CD68/MMP12 double-positive ratio,^47^ while the other measured intraplaque MMP8, a marker also expressed by neutrophils.^48^ Our module-score approach therefore provides the first direct transcriptional evidence that total macrophages and certain distinct macrophage programmes associate with secondary cardiovascular risk.

Our data demonstrate that plaque macrophages can be divided into four archetypes: inflammatory macrophages, resident-like LAMs, LAMs, and iLAMs (*Figure 1E*). While it has been shown previously^15^ that iLAM content correlates with symptomatic disease at the time of surgery and we could replicate this (*Figure 1D*; Supplementary data online, *Figure S6F*), we now show that specific iLAM marker expression is also predictive of MACE after a 3-year or 10-year follow-up period (*Figure 4FG*). Notably, some LAM markers, FABP5 and CTSL but not TREM2, were also reproducibly predictive of MACE (*Figure 4G*), whereas several inflammatory and resident-like LAM markers did associate in our AE cohort, but could not be reproduced in CPIP. The iLAM markers that were associated in both cohorts hint towards links with macrophage metabolism, stress responses, and tissue remodelling. HK2 and FBP1 are key metabolic enzymes regulating glycolysis and gluconeogenesis, respectively, influencing macrophage energy balance and polarization.^49^ PLIN2 and G0S2 are involved in lipid droplet formation and lipolysis,^50,51^ linking macrophage lipid handling to foam cell formation. ERO1α participates in oxidative protein folding and endoplasmic reticulum stress responses, while adrenomedullin and Vascular Endothelial Growth Factor A (VEGFA) are proangiogenic and hypoxia-responsive factors that can affect plaque neovascularization and stability.^52–54^ Finally, MMP19 encodes a matrix metalloproteinase contributing to extracellular matrix remodelling, which may be affecting plaque stability.^55^ Together, these genes highlight interconnected metabolic, inflammatory, and structural pathways shaping macrophage function in atherosclerotic lesions.

Canonical iLAM marker *TREM1*,^15^ which also associates with symptoms at surgery and MACE at follow-up in our data (*Figures 1D* and *4G*), has also been implicated in female enriched populations in a study of sex differences in atherosclerosis progression,^56^ further demonstrating the adverse nature of iLAMs in atherosclerotic plaques. Nonetheless, also TREM2-enriched LAM populations, which have generally been linked to a more favourable phenotype,^57–59^ have been linked to an adverse outcome, i.e. aortic aneurysm formation,^60^ by promoting endothelial dysfunction. This is in line with the LAM module (*Figure 4E* and *F*) and individual LAM genes (*Figure 4G*) also associating with MACE at follow-up in our data, albeit less prominently than the iLAMs, and suggests that the exact role of different macrophage types is heavily context and timing dependent in a complex system such as atherosclerosis.

Our data are in line with previously identified macrophage subtypes. For example, inflammatory population C4 has an IFNIC signature, and LAM population C5 is MAC^AIR^-like^61^ (see Supplementary data online, *Table S1*).

Interestingly, all the resident-like LAMs and LAM populations share a common gene expression signature hinting at a shared origin. This includes expression of *PLTP*, a phospholipid transfer protein that has been mostly negatively implicated in atherosclerosis through its effects on cholesterol efflux, lipoprotein metabolism, and inflammation,^62,63^ leading to cholesterol accumulation. The effect of macrophage-specific *PLTP* expression in an atherosclerotic setting, however (i.e. adverse or beneficial), remains controversial.^63–66^ A possible answer to this discrepancy involves the difference between systemic and local plaque macrophage *PLTP* expression. That is, loss of systemic PLTP expression leads to cholesterol accumulation in the circulation and consequently plaque progression, while locally produced macrophage-derived PLTP rescues this phenotype and reduces plaque progression.^64,67^ Intriguingly, the resident-like LAM population C7 expresses some markers commonly associated with tissue resident macrophages, such as *FOLR2* and *MRC1*, but mostly overlaps in gene expression with LAM populations C5 and C6, except for a distinctive lack of *TREM2* expression. This could be due to a longer term ‘settling in’ of monocyte-derived macrophages, where a subset loses *TREM2* and gains resident marker expression and becomes more anti-inflammatory, instead of directly developing into full-blown iLAMs. It might be a promising avenue for future research to work out the molecular trigger behind this trajectory deviation and find a way to therapeutically guide plaque macrophages to a more favourable, anti-inflammatory *TREM2*-low resident-like LAM state.

The iLAM populations (C8, C9, C10) divide into *OLR1^hi^*, *ABCA1^hi^*, and hypoxic- and lipid-stress-induced apoptotic and necrotic iLAMs. Correspondingly, the iLAM populations were also the most difficult to sequence, as shown by their relatively low number (*Figure 1E*), absence in several external datasets (see Supplementary data online, *Figure S2A*), and less diverse individual patient contributions (see Supplementary data online, *Figure S1F*). The death-spiral in which these foamy cells are trapped may be linked to their loss of *TREM2* expression hampering regulation of cholesterol uptake.^57–59,68^ Interestingly, mostly the iLAM populations and adjacent cells seem to be enriched for pathways and TF networks involved in cellular proliferation, suggesting that lipid exposure triggers macrophage expansion, in line with previous data.^69,70^ Of note, TGFβ and SALL1 signalling, active in iLAMs, were also shown to be important for the establishment of the microglia phenotype.^71^

In mice, it has been shown that it is mostly the classical (Ly6C^high^) monocytes that enter the lesion and turn into inflammatory macrophages.^72–75^ In our human data, the inflammatory macrophages also show a clear pattern of non-classical monocyte gene expression (e.g. *FCGR3^hi^* and *CX3CR1^hi^*), which is corroborated by the trajectory analyses, which trace the lineage of the inflammatory macrophages via the non-classical monocytes and our *in vitro* validations. This may indicate clear mouse-man differences where human atherosclerosis develops slowly over years involving recruitment of both monocyte subtypes, whereas murine atherosclerosis is more acute, triggering only classical monocyte activation.

Next to monocytes and tissue resident cells, a fraction of plaque macrophages may originate from smooth muscle cells (SMCs).^76^ In our original 18-patient cohort,^16^ we indeed identified a subset of foam cells with SMC marker expression. However, in the full cohort, no such subpopulation was detected. Trajectory analyses on the full set of cell types also failed to show a link between the SMC populations and the macrophages. This suggests that either SMC-derived macrophages in human lesions are rarer or more fragile, such as foam cells, lost during the prep procedure. Additionally, the 10× libraries were prepared from CD45 sorted cells, which may have limited the detection of the CD45-low SMCs.

Collectively, our plaque and PBMC data demonstrate the importance of influx of blood monocytes as an important determinant of the abundant presence of macrophages in the atherosclerotic plaque. This is supported by the main cellular differentiation trajectory, which starts with the monocytes differentiating into inflammatory macrophages before ending up as (i)LAMs. Furthermore, lack of cell division and proliferation pathway and gene activity in the inflammatory macrophages, in addition to the continued expression of monocyte markers in most plaque macrophages, support a major role for recruited cells in plaques.

### Limitations of the study

Immune cell composition of human plaques and contribution to clinical events may be disease stage dependent.^77^ However, since our cohort consists predominantly of advanced symptomatic plaques, we could not directly confirm this in the present study.

Based on only transcriptomic data, we could not assess which triggers drive inflammatory plaque macrophages to turn into LAMs first, or directly into iLAMs. This is likely decided by variable exposures in the plaque micro-environment, but spatial (transcriptomic) information is needed to resolve this.

Our scRNA-seq cohort of 46 patients does not have enough power to directly associate phenotypical patient traits with cell type abundance. However, we could robustly make such associations by deploying a data deconvolution approach to two large and independent bulk RNA-seq cohorts.

## Conclusions

In conclusion, we provide a detailed characterization of human plaque macrophages, their differentiation paths, and their associations with secondary clinical events. Most interestingly, we identify a potential prognostic value of plaque macrophage abundance and iLAM marker expression for adverse outcomes, underscoring the critical role of macrophage biology in human atherosclerosis.

## Supplementary Material

ehag117_Supplementary_Data

## Declarations

### Data Availability

All single-cell RNA-seq data used in this study have been deposited at doi:10.34894/TYHGEF, doi.org/10.34894/DDYKLL, GSE210152, GSE155512, and GSE131780.

All R and Python scripts used to perform the analyses presented in this study have been deposited at Zenodo (DOI 10.5281/zenodo.13740522).
