# Supplementary material for: Carotid plaque macrophage burden and inflammatory lipid-associated macrophage markers predict secondary major adverse cardiovascular events after endarterectomy
Source: Eur Heart J. 2026 Feb 27;47(28):3821–36. doi: 10.1093/eurheartj/ehag117 (PMC13384728; doi:10.1093/eurheartj/ehag117)
Supplement: ehag117_Supplementary_Data [file ehag117_supplementary_data.zip › Supplemental Figures.pdf]

Sup. Figure 1

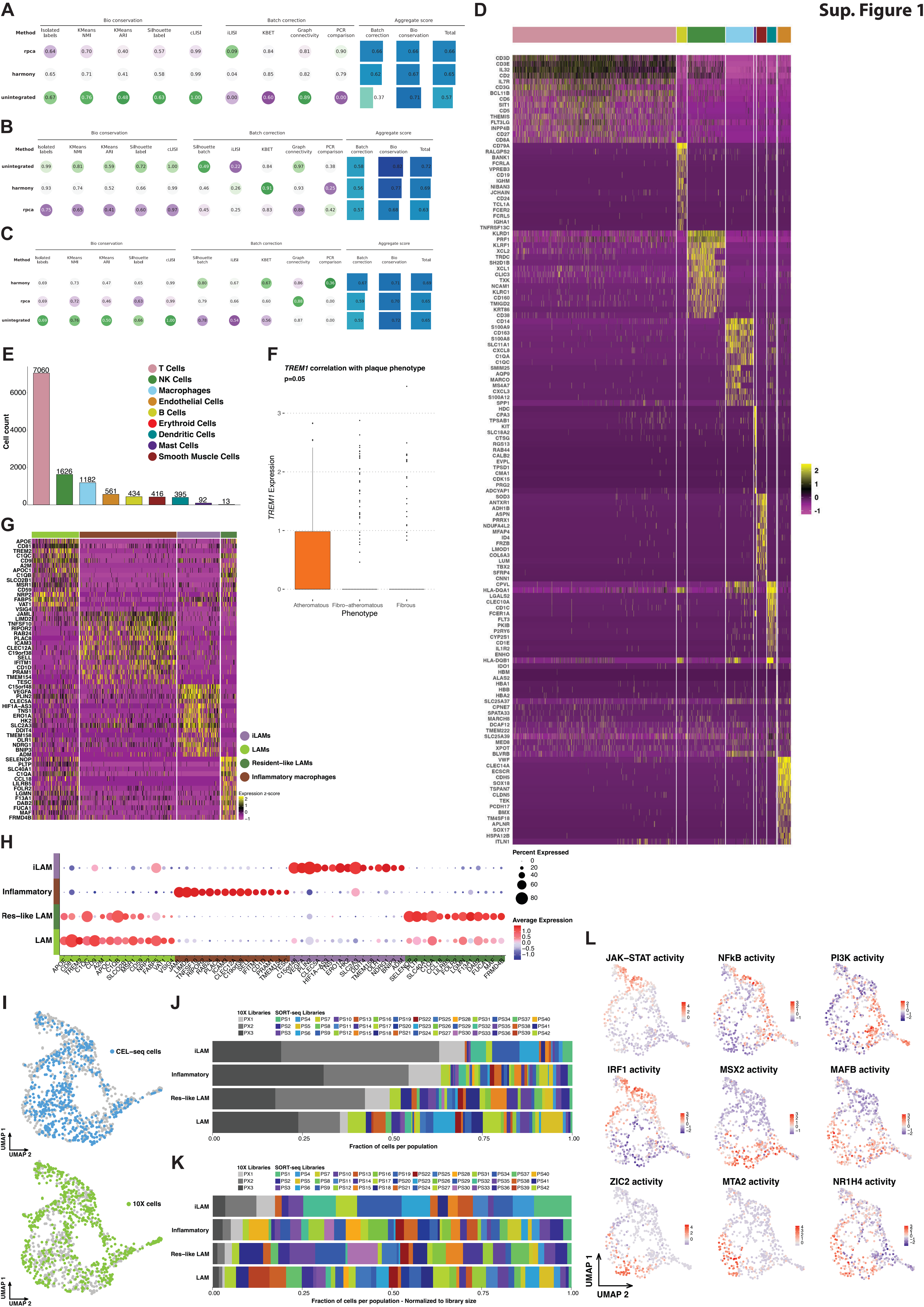

A
Bio conservation
Batch correction
Aggregate score
Method
Isolated labels
KMeans NMI
KMeans ARI
Silhouette label
cLISI
iLISI
KBET
Graph connectivity
PCR comparison
Batch correction
Bio conservation
Total
rpca 0.64 0.70 0.40 0.57 0.99 0.09 0.84 0.81 0.90 0.66 0.66 0.66
harmony 0.65 0.71 0.41 0.58 0.99 0.04 0.85 0.82 0.79 0.62 0.67 0.65
unintegrated 0.67 0.76 0.48 0.63 1.00 0.00 0.60 0.89 0.00 0.37 0.71 0.57
B
Silhouette batch
unintegrated 0.99 0.81 0.59 0.72 1.00 0.49 0.22 0.84 0.97 0.38 0.58 0.82 0.72
harmony 0.93 0.74 0.52 0.66 0.99 0.46 0.26 0.91 0.93 0.25 0.56 0.77 0.69
rpca 0.75 0.65 0.41 0.60 0.97 0.45 0.25 0.83 0.88 0.42 0.57 0.68 0.63
C
harmony 0.69 0.73 0.47 0.65 0.99 0.80 0.67 0.67 0.86 0.36 0.67 0.71 0.69
rpca 0.69 0.72 0.46 0.63 0.99 0.79 0.66 0.60 0.88 0.00 0.59 0.70 0.65
unintegrated 0.69 0.76 0.50 0.66 1.00 0.78 0.54 0.56 0.87 0.00 0.55 0.72 0.65
D
E
Cell count
7060 1626 1182 561 434 416 395 92 13
T Cells
NK Cells
Macrophages
Endothelial Cells
B Cells
Erythroid Cells
Dendritic Cells
Mast Cells
Smooth Muscle Cells
F
TREM1 correlation with plaque phenotype
p=0.05
TREM1 Expression
Atheromatous
Fibro-atheromatous
Fibrous
Phenotype
G
iLAMs
LAMs
Resident-like LAMs
Inflammatory macrophages
Expression z-score
H
iLAM
Inflammatory
Res-like LAM
LAM
Percent Expressed
Average Expression
I
CEL-seq cells
10X cells
UMAP 1
UMAP 2
J
10X Libraries
SORT-seq Libraries
Fraction of cells per population
K
Fraction of cells per population - Normalized to library size
L
JAK-STAT activity
NFkB activity
PI3K activity
IRF1 activity
MSX2 activity
MAFB activity
ZIC2 activity
MTA2 activity
NR1H4 activity

Sup. Figure 2

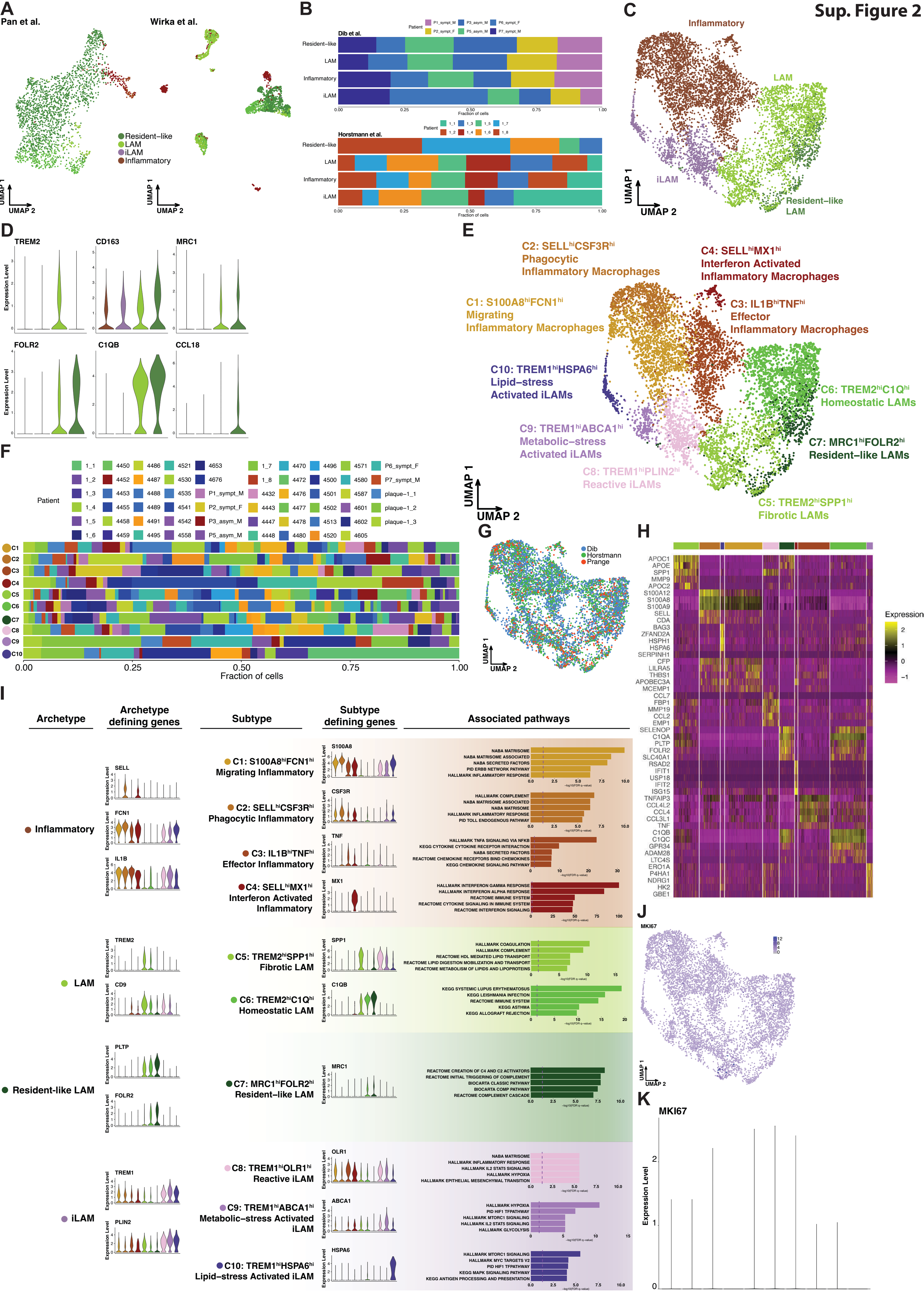

A
Pan et al.
Wirka et al.
Resident–like
LAM
iLAM
Inflammatory
UMAP 1
UMAP 2
B
Dib et al.
Patient
P1_sympt_M
P2_sympt_F
P3_asym_M
P5_asym_M
P6_sympt_F
P7_sympt_M
Resident–like
LAM
Inflammatory
iLAM
Fraction of cells
Horstmann et al.
C
Inflammatory
LAM
iLAM
Resident–like LAM
D
TREM2
CD163
MRC1
FOLR2
C1QB
CCL18
Expression Level
E
C2: SELL^hi CSF3R^hi Phagocytic Inflammatory Macrophages
C4: SELL^hi MX1^hi Interferon Activated Inflammatory Macrophages
C1: S100A8^hi FCN1^hi Migrating Inflammatory Macrophages
C3: IL1B^hi TNF^hi Effector Inflammatory Macrophages
C10: TREM1^hi HSPA6^hi Lipid–stress Activated iLAMs
C6: TREM2^hi C1Q^hi Homeostatic LAMs
C9: TREM1^hi ABCA1^hi Metabolic–stress Activated iLAMs
C7: MRC1^hi FOLR2^hi Resident–like LAMs
C8: TREM1^hi PLIN2^hi Reactive iLAMs
C5: TREM2^hi SPP1^hi Fibrotic LAMs
F
Patient
Fraction of cells
G
Dib
Horstmann
Prange
H
Expression
I
Archetype
Archetype defining genes
Subtype
Subtype defining genes
Associated pathways
Inflammatory
C1: S100A8^hi FCN1^hi Migrating Inflammatory
C2: SELL^hi CSF3R^hi Phagocytic Inflammatory
C3: IL1B^hi TNF^hi Effector Inflammatory
C4: SELL^hi MX1^hi Interferon Activated Inflammatory
LAM
C5: TREM2^hi SPP1^hi Fibrotic LAM
C6: TREM2^hi C1Q^hi Homeostatic LAM
Resident-like LAM
C7: MRC1^hi FOLR2^hi Resident–like LAM
iLAM
C8: TREM1^hi OLR1^hi Reactive iLAM
C9: TREM1^hi ABCA1^hi Metabolic–stress Activated iLAM
C10: TREM1^hi HSPA6^hi Lipid–stress Activated iLAM
J
MKI67
K
MKI67
Expression Level

Sup. Figure 3

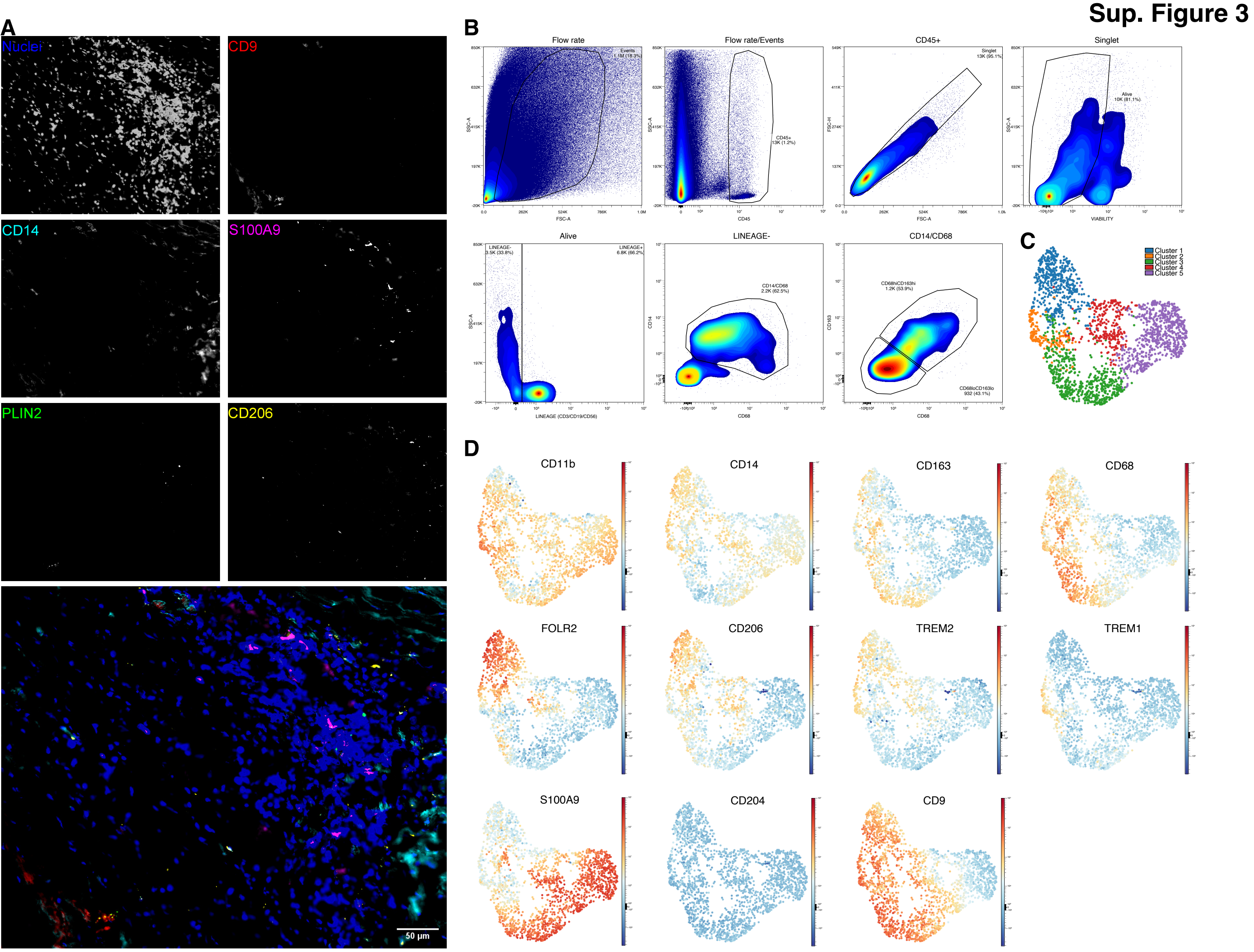
A
Nuclei
CD9
CD14
S100A9
PLIN2
CD206
50 µm
B
Flow rate
Flow rate/Events
CD45+
Singlet
Alive
LINEAGE-
CD14/CD68
C
Cluster 1
Cluster 2
Cluster 3
Cluster 4
Cluster 5
D
CD11b
CD14
CD163
CD68
FOLR2
CD206
TREM2
TREM1
S100A9
CD204
CD9

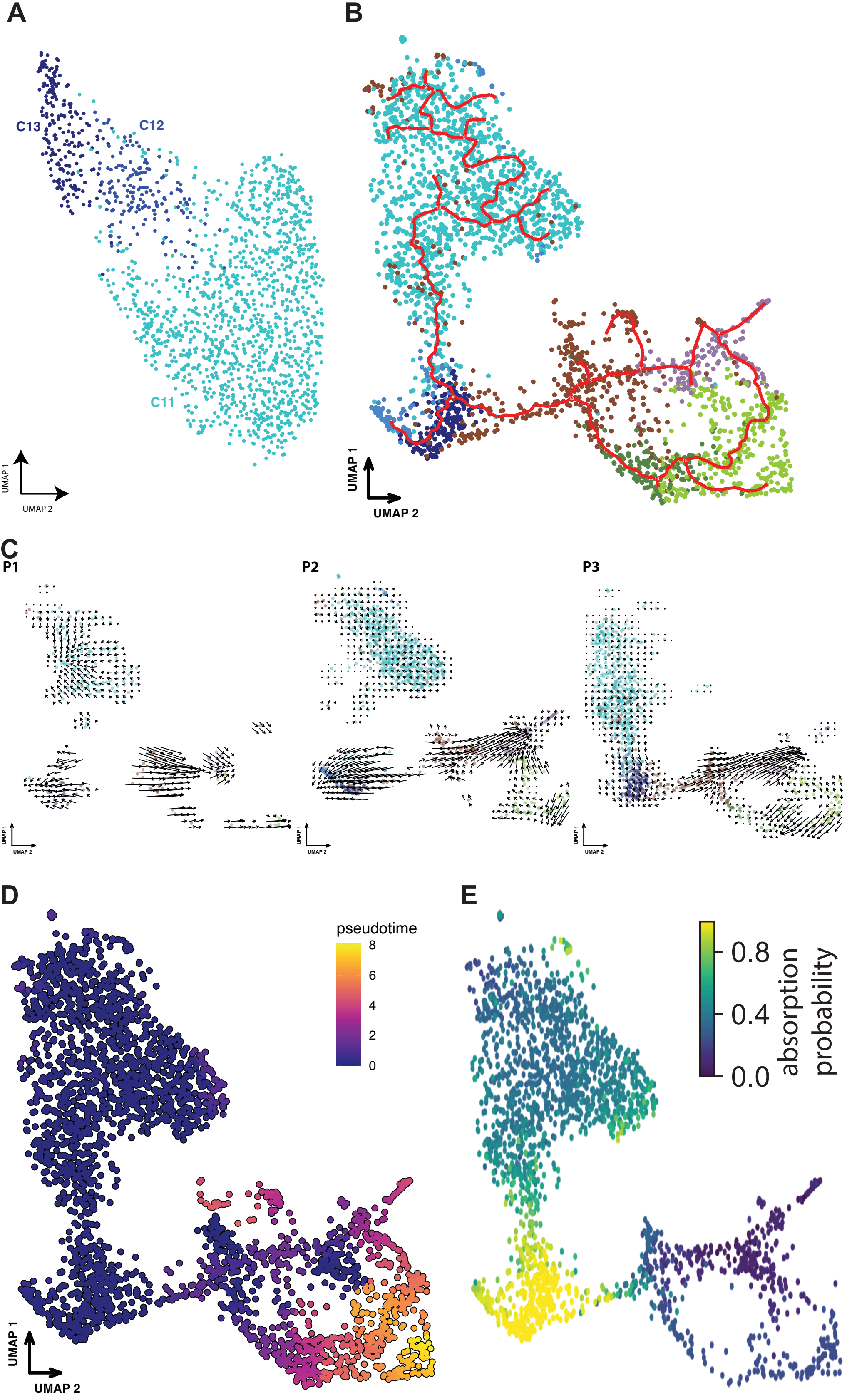
A
C13
C12
C11
UMAP 1
UMAP 2
B
UMAP 1
UMAP 2
C
P1
P2
P3
D
pseudotime
8
6
4
2
0
UMAP 1
UMAP 2
E
0.8
0.4
0.0
absorption probability

Sup. Figure 5

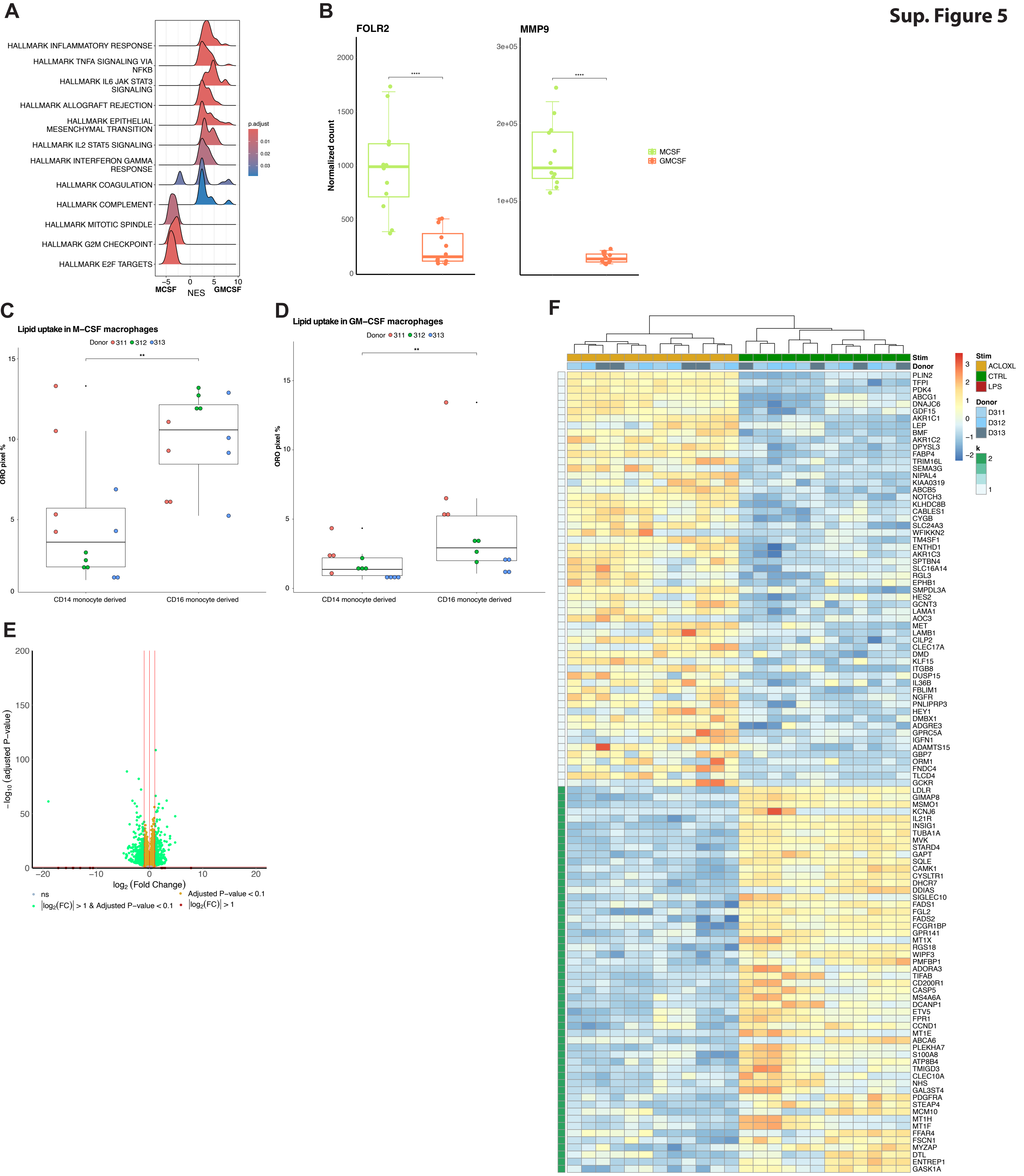
A
HALLMARK INFLAMMATORY RESPONSE
HALLMARK TNFA SIGNALING VIA NFKB
HALLMARK IL6 JAK STAT3 SIGNALING
HALLMARK ALLOGRAFT REJECTION
HALLMARK EPITHELIAL MESENCHYMAL TRANSITION
HALLMARK IL2 STAT5 SIGNALING
HALLMARK INTERFERON GAMMA RESPONSE
HALLMARK COAGULATION
HALLMARK COMPLEMENT
HALLMARK MITOTIC SPINDLE
HALLMARK G2M CHECKPOINT
HALLMARK E2F TARGETS
p.adjust
0.01
0.02
0.03
−5
0
5
10
MCSF
NES
GMCSF
B
FOLR2
MMP9
Normalized count
2000
1500
1000
500
0
3e+05
2e+05
1e+05
****
MCSF
GMCSF
C
Lipid uptake in M−CSF macrophages
Donor 311 312 313
ORO pixel %
15
10
5
0
**
CD14 monocyte derived
CD16 monocyte derived
D
Lipid uptake in GM−CSF macrophages
Donor 311 312 313
ORO pixel %
**
CD14 monocyte derived
CD16 monocyte derived
E
200
150
100
50
0
$-\log_{10}$ (adjusted P−value)
−20
−10
0
10
20
$\log_2$ (Fold Change)
ns
Adjusted P−value < 0.1
$|\log_2(FC)| > 1$ & Adjusted P−value < 0.1
$|\log_2(FC)| > 1$
F
Stim
Donor
Stim
ACLOXL
CTRL
LPS
Donor
D311
D312
D313
k
2
1
3
2
1
0
−1
−2
PLIN2
TFPI
PDK4
ABCG1
DNAJC6
GDF15
AKR1C1
LEP
BMF
AKR1C2
DPYSL3
FABP4
TRIM16L
SEMA3G
NIPAL4
KIAA0319
ABCB5
NOTCH3
KLHDC8B
CABLES1
CYGB
SLC24A3
WFIKKN2
TM4SF1
ENTHD1
AKR1C3
SPTBN4
SLC16A14
RGL3
EPHB1
SMPDL3A
HES2
GCNT3
LAMA1
AOC3
MET
LAMB1
CILP2
CLEC17A
DMD
KLF15
ITGB8
DUSP15
IL36B
FBLIM1
NGFR
PNLIPRP3
HEY1
DMBX1
ADGRE3
GPRC5A
IGFN1
ADAMTS15
GBP7
ORM1
FNDC4
TLCD4
GCKR
LDLR
GIMAP8
MSMO1
KCNJ6
IL21R
INSIG1
TUBA1A
MVK
STARD4
GAPT
SQLE
CAMK1
CYSLTR1
DHCR7
DDIAS
SIGLEC10
FADS1
FGL2
FADS2
FCGR1BP
GPR141
MT1X
RGS18
WIPF3
PMFBP1
ADORA3
TIFAB
CD200R1
CASP5
MS4A6A
DCANP1
ETV5
FPR1
CCND1
MT1E
ABCA6
PLEKHA7
S100A8
ATP8B4
TMIGD3
CLEC10A
NHS
GAL3ST4
PDGFRA
STEAP4
MCM10
MT1H
MT1F
FFAR4
FSCN1
MYZAP
DTL
ENTREP1
GASK1A

Sup Figure 6

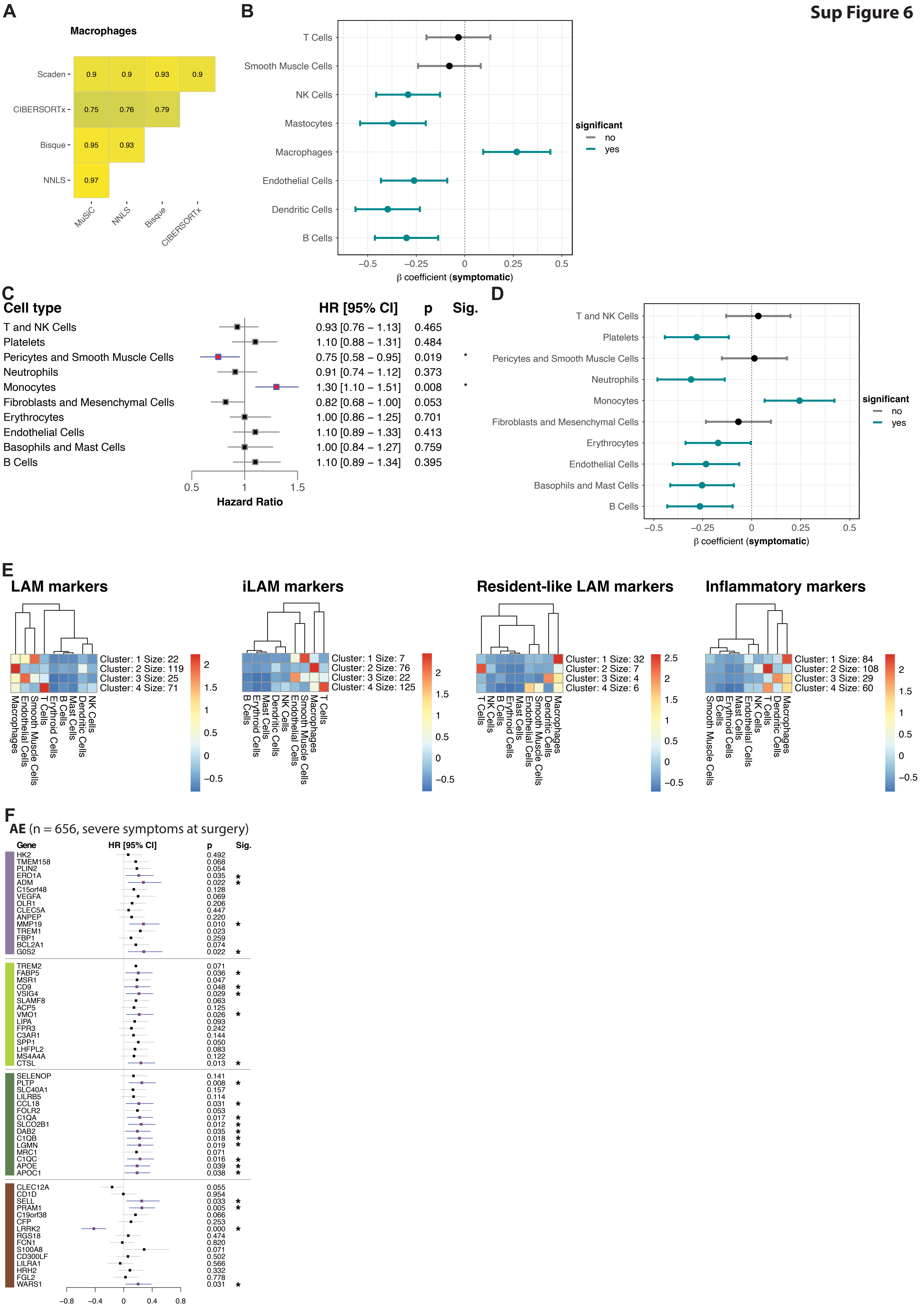
A
Macrophages
Scaden 0.9 0.9 0.93 0.9
CIBERSORTx 0.75 0.76 0.79
Bisque 0.95 0.93
NNLS 0.97
MuSiC
NNLS
Bisque
CIBERSORTx
B
T Cells
Smooth Muscle Cells
NK Cells
Mastocytes
Macrophages
Endothelial Cells
Dendritic Cells
B Cells
−0.5 −0.25 0 0.25 0.5
β coefficient (symptomatic)
significant
no
yes
C
Cell type
HR [95% CI]
p
Sig.
T and NK Cells 0.93 [0.76 – 1.13] 0.465
Platelets 1.10 [0.88 – 1.31] 0.484
Pericytes and Smooth Muscle Cells 0.75 [0.58 – 0.95] 0.019 *
Neutrophils 0.91 [0.74 – 1.12] 0.373
Monocytes 1.30 [1.10 – 1.51] 0.008 *
Fibroblasts and Mesenchymal Cells 0.82 [0.68 – 1.00] 0.053
Erythrocytes 1.00 [0.86 – 1.25] 0.701
Endothelial Cells 1.10 [0.89 – 1.33] 0.413
Basophils and Mast Cells 1.00 [0.84 – 1.27] 0.759
B Cells 1.10 [0.89 – 1.34] 0.395
0.5 1 1.5
Hazard Ratio
D
T and NK Cells
Platelets
Pericytes and Smooth Muscle Cells
Neutrophils
Monocytes
Fibroblasts and Mesenchymal Cells
Erythrocytes
Endothelial Cells
Basophils and Mast Cells
B Cells
−0.5 −0.25 0 0.25 0.5
β coefficient (symptomatic)
significant
no
yes
E
LAM markers
Cluster: 1 Size: 22
Cluster: 2 Size: 119
Cluster: 3 Size: 25
Cluster: 4 Size: 71
Macrophages
Endothelial Cells
Smooth Muscle Cells
T Cells
Erythroid Cells
B Cells
Mast Cells
Dendritic Cells
NK Cells
iLAM markers
Cluster: 1 Size: 7
Cluster: 2 Size: 76
Cluster: 3 Size: 22
Cluster: 4 Size: 125
B Cells
Erythroid Cells
Mast Cells
Dendritic Cells
NK Cells
Endothelial Cells
Smooth Muscle Cells
Macrophages
T Cells
Resident-like LAM markers
Cluster: 1 Size: 32
Cluster: 2 Size: 7
Cluster: 3 Size: 4
Cluster: 4 Size: 6
T Cells
NK Cells
B Cells
Erythroid Cells
Mast Cells
Endothelial Cells
Smooth Muscle Cells
Dendritic Cells
Macrophages
Inflammatory markers
Cluster: 1 Size: 84
Cluster: 2 Size: 108
Cluster: 3 Size: 29
Cluster: 4 Size: 60
Smooth Muscle Cells
B Cells
Erythroid Cells
Mast Cells
Endothelial Cells
NK Cells
T Cells
Dendritic Cells
Macrophages
F
AE (n = 656, severe symptoms at surgery)
Gene
HR [95% CI]
p
Sig.
HK2 0.492
TMEM158 0.068
PLIN2 0.054
ERO1A 0.035 *
ADM 0.022 *
C15orf48 0.128
VEGFA 0.069
OLR1 0.206
CLEC5A 0.447
ANPEP 0.220
MMP19 0.010 *
TREM1 0.023
FBP1 0.259
BCL2A1 0.074
G0S2 0.022 *
TREM2 0.071
FABP5 0.036 *
MSR1 0.047
CD9 0.048 *
VSIG4 0.029 *
SLAMF8 0.063
ACP5 0.125
VMO1 0.026 *
LIPA 0.093
FPR3 0.242
C3AR1 0.144
SPP1 0.050
LHFPL2 0.083
MS4A4A 0.122
CTSL 0.013 *
SELENOP 0.141
PLTP 0.008 *
SLC40A1 0.157
LILRB5 0.114
CCL18 0.031 *
FOLR2 0.053
C1QA 0.017 *
SLCO2B1 0.012 *
DAB2 0.035 *
C1QB 0.018 *
LGMN 0.019 *
MRC1 0.071
C1QC 0.016 *
APOE 0.039 *
APOC1 0.038 *
CLEC12A 0.055
CD1D 0.954
SELL 0.033 *
PRAM1 0.005 *
C19orf38 0.066
CFP 0.253
LRRK2 0.000 *
RGS18 0.474
FCN1 0.820
S100A8 0.071
CD300LF 0.502
LILRA1 0.566
HRH2 0.332
FGL2 0.778
WARS1 0.031 *
−0.8 −0.4 0 0.4 0.8

Supplemental Figure 1. Plaque macrophages can be divided in 4 archetypes. A) Table of SCIB metrics for integration of all libraries by batch correcting for sequencing method. SCIB = Single Cell Integration Benchmarking. B) Table of SCIB metrics for integration of all SORT-seq libraries by batch correcting per patient. C) Table of SCIB metrics for integration of all 10X libraries by batch correcting per patient D) Heatmap of top 15 marker genes per population. E) Barplot showing the absolute abundance of cell types. F) Barplot showing normalized *TREM1* expression in macrophages of patients binned per plaque phenotype. Expression data from 46 patients scRNA-seq. P-value is of a Kruskal-Wallis rank sum test of the correlation between phenotype and gene expression. G-H) Heatmap and dotplot of top 15 marker genes per macrophage archetype. Overlapping markers are plotted only once. I) UMAPs colored by library preparation method. Top: CEL-seq cells. Bottom: 10X cells. J) Barplot of relative cell contribution of each patient to each population. K) Barplot of relative cell contribution of each patient to each population, normalized to library size per patient. L) UMAPs of plaque macrophages showing pathway or TF network activity. Scale: z-score of activity

Supplemental Figure 2. Integration with external datasets confirms macrophage subpopulations

A) UMAPs showing macrophages from Pan et al.(68) (left) and Wirka et al.(69) (right) reference-mapped to our archetypes. B) Barplot of relative cell contribution of each patient to each reference-mapped archetype normalized to library size per patient. Top: Dib et al. data. Bottom: Horstmann et al. data. C) UMAP of integrated plaque macrophages, colored by reference-mapped archetype. D) Violin plots of resident-like / LAM marker gene expression. E) UMAP of integrated plaque macrophages colored by subpopulation. F) Barplot of relative cell contribution of each patient to each population normalized to library size per patient. G) UMAP of integrated plaque macrophages, colored by dataset of origin. H) Heatmap of top 5 markers per macrophage population. I) Violin plots of marker genes on the archetype (left) and subpopulation (middle) level. Bar plots (right) show top 5 GSEA pathway enrichment per population. FDR = False Discovery Rate. J) UMAP and K) violin plot of MKI67 expression in the integrated plaque macrophages.

Supplemental Figure 3: Macrophage archetype presence in human carotid atherosclerotic plaques could be confirmed by fluorescent immunohistochemistry and spectral flow cytometry for key marker proteins.

A) Multispectral immunohistochemistry of human carotid atherosclerotic plaque tissue. Top 6 panels: individual channels stained for nuclei, CD9, CD14, S100A9, PLIN2, and CD206. Bottom: composite image. Scale bar represents 50 µm. B) FACS gating strategy. C) UMAP visualization of gated CD14/CD68 population, colored by FlowSOM defined clusters. D) UMAP visualization of gated CD14/CD68 populations. Events are colored by staining intensity for the indicated antibody.

Supplemental Figure 4. Plaque macrophages converge into iLAMs as terminal cell type.

A) UMAP of PBMC-derived monocytes. B) UMAP of all macrophage and monocyte populations overlayed with cellular trajectory as defined by monocle3.

C) UMAPs with RNA velocity vectors (arrows) for 10X library P1 (left), P2 (middle), and P3 (right). D) UMAP of all macrophage and monocyte populations scored by pseudotime rooted in the classical monocyte population as calculated by monocle3. E) UMAP of all macrophage

and monocyte populations showing non-classical monocyte lineage commitment per cell as defined by CellRank. Scale: absorption probability.

Supplemental Figure 5. Macrophage differentiation axes can be recapitulated in vitro. A) GSEA of HALLMARK pathways in M-CSF vs. GM-CSF derived macrophages. B) RNA-seq data of *FOLR2* and *MMP9* gene expression in M-CSF vs. GM-CSF derived macrophages. C) Boxplot showing the proportion of pixels colored by oil-red-o (ORO) in acLDL-stimulated M-CSF macrophages. D) Boxplot showing the proportion of pixels colored by oil-red-o (ORO) in acLDL-stimulated GM-CSF macrophages. E) Volcanoplot of differentially expressed genes in lipid laden vs. DMSO control macrophages. F) Heatmap of top 50 differentially expressed genes in lipid laden vs. DMSO control macrophages. Scale = z-score. DMSO = dimethylsulfoxide. M-CSF = Macrophage Colony Stimulating Factor. GM-CSF = Granulocyte Macrophage Colony Stimulating Factor. acLDL = acetylated low-density lipoprotein. oxLDL = oxidized low-density lipoprotein. LPS = lipopolysaccharide. Statistical differences were tested by one-way ANOVA with Tukey's HSD post-hoc test for pairwise comparisons. ns = not significant; * = padj < 0.05; ** = padj < 0.01; *** = padj < 0.001; **** = padj < 0.0001.

Supplemental Figure 6. Human plaque macrophage content is associated with MACE. A) Correlation matrix of macrophage proportion per deconvolution algorithm. B) Forest plot showing ß-coefficient for the association between the presence of cerebrovascular event (Stroke or TIA) prior to carotid endarterectomy and various cell types at the time of surgery. Green color denotes a significant association (p < 0.05). C) Forest plot showing the Hazard Ratio for association of MACE with various cell types at 3-year follow-up. Cell types based on Athero Express bulk RNA-seq data deconvoluted using the Tabula Sapiens reference dataset. Red boxes and blue lines denote a significant association (p < 0.05). D) Forest plot showing ß-coefficient for the association between presence of cerebrovascular event (CVA or TIA) prior to carotid endarterectomy and various cell types at the time of surgery. Cell types based on Athero Express bulk RNA-seq data deconvoluted using the Tabula Sapiens reference dataset. Green color denotes a significant association (p < 0.05). E) *k*-means clustered heatmaps of archetype marker expression in all cell types. Average expression = z-scaled per gene. F) Forest plot showing correlation of individual marker gene expression values and presence of cerebrovascular event (Stroke or TIA) prior to carotid endarterectomy. AE = Athero Express HR = Hazard Ratio. CI = Confidence Interval.
