# Supplementary material for: Carotid plaque macrophage burden and inflammatory lipid-associated macrophage markers predict secondary major adverse cardiovascular events after endarterectomy
Source: Eur Heart J. 2026 Feb 27;47(28):3821–36. doi: 10.1093/eurheartj/ehag117 (PMC13384728; doi:10.1093/eurheartj/ehag117)
Supplement: ehag117_Supplementary_Data [file ehag117_supplementary_data.zip › Supplemental Methods.docx]

### Patients and scRNA-seq library construction

This study complies with the Declaration of Helsinki and all participants provided informed consent at University Medical Center, Utrecht, The Netherlands, St. Antonius Hospital, Nieuwegein, The Netherlands, and University Hospital of Skåne, Lund/Malmö, Sweden. The local medical ethical committees of all involved institutes approved these studies. Patients were included from the Athero Express cohort as described(1). SORT-seq libraries were constructed as described(1). 10X-genomics libraries were constructed from CD45^+^ sorted cells as described(2). Blood for PBMC libraries was drawn 24 h before endarterectomy.

### Software

All analyses were performed using custom R (version 4.2.2) and Python (version 3.9.15) scripts. scRNA-seq data handling and analyses were performed with Seurat(3) (v4.2.0) and scib-metrics(4) (v0.5.0), and visualised with ggplot2(5) (v3.4.0) and ggpubr(6) (v0.4.0). Bulk RNA-seq deconvolution was performed with Scaden(7) (v1.1.2). Pathway and network enrichment was calculated with EGSEA(8) (v1.24.0), AUCell v(1.126.0), and decoupleR(9) (v1.3.1). Trajectory and cell fate analyses were performed with monocle3(10, 11) v(1.2.9), velocyto.R(12) (v0.6), and CellRank(13) (v1.5.1). Differential gene expression was computed using DESeq2(14). Module scores were calculated using GSVA(15) . Drug-gene interactions were retrieved using DGIdb(16) (rDGIdb v.1.22.0). Oil-red-O stainings were quantified with QuPath (v0.5.1).

Cartoons, schematics, and panel layouts were created with BioRender.com and Adobe Illustrator (v27.0.1).

### Bioinformatic analyses

Fully commented R code covering the whole project is available at Zenodo. Briefly, per sample count tables were loaded as individual Seurat objects, normalized using SCTransform, and filtered. (Long) noncoding (RNA) genes, and cells with less than 200 or more than 5000 genes, mitochondrial reads > 5%, and apoptotic marker reads > 2% were removed. Cell cycle, % mt, % apoptotic marker genes *such as KCNQ1OT1*, *UGDH-AS1*, and *GHET1* were regressed out. Subsequently, SORT-seq and microfluidics-based human plaque libraries were integrated using Seurat reciprocal PCA integration to correct for batch effects caused by the different library creation methods. This yielded a good overall integration score based on metrics such as kBET(17) and iLISI(18) as computed by the scib-metrics package(4), while conserving biological variance as measured by e.g., cLISI(18) (Supplemental Figure 1A). Integrating individual patient samples yielded only marginal improvements in aggregate integration score and was thus omitted to maximize biological variance conservation. (Supplemental Figure 1BC). To further assess the robustness of the integration, we directly compared RPCA against Harmony integration(18), which showed comparable results (Supplemental Figure 1ABC).

Lastly, PCA and UMAP reductions were calculated, and clusters were called using 30 PCA dimensions and a resolution of 1.25.

To subset macrophages from the full scRNA-seq dataset, we mapped our integrated 10X and SORT-seq data to a PBMC reference dataset(19) to obtain a comprehensive overview of population identities, made a subset of plaque myeloid cells, and filtered out the cells classified as dendritic cells. Lastly, we enriched for macrophages by only keeping cells lacking expression of the non-macrophage markers *CD3E*, *NCAM1*, and *CD79B*.

Pathway and transcription factor network enrichment of single cells in the four macrophage subsets was performed with decoupleR(9).

Virtual lineage tracing was performed with Monocle3, Velocyto, and CellRank following standard workflows. Briefly, we used the quantified divergence of transcriptomes between neighboring cells to construct a most likely trajectory for cellular development or differentiation using monocle3. Next, we called pseudotime along this trajectory using the classical monocyte population as root. We then calculated RNA velocity vectors using deltaT = 1, kCells = 25, and fit.quantile = 0.02, and plotted them on the monocyte / macrophage UMAP with n= 200 (75 for P1), grid.n = 40, and nPCs = 30. Lastly, we used generalized perron cluster cluster Analysis(20) (G-PCCA) to classify a Markov chain of cell-cell transition probabilities (based on pseudotime and RNA velocity) into likely terminal states using CellRank. We then calculated the absorption probability of each individual transient cell towards a terminal state. I.e., we identified clusters of cells representing likely cellular end-states and then calculated each cell's probability to obtain this state in the future.

Geneset enrichment analyses of (G)M-CSF signatures was performed with AUCell. (G)M-CSF treated macrophage differentially expressed genes were obtained from GSE99056. Genes with padj < 0.05 and |lfc| > 2 compared to naïve macrophages were selected. Enrichment in the single cell macrophage populations was calculated using the AUCell standard workflow.

**Deconvolution reference (Tabula Sapiens)**

The Tabula Sapiens version 1 was utilised, encompassing tissues and organs from 15 donors. To reduce the complexity, the cells were selected and consolidated into 10 major cell types based on the expected presence in the atherosclerotic plaques - basophil/mast cell, B cell, endothelial cell, erythrocyte, mesenchymal cell/fibroblast, monocyte, neutrophil, platelet, pericyte/SMC, and T/NK cell. Thereafter, a total of 1,000 randomly selected cells from each major cell type in the chosen grouping were included in the final scRNA-seq reference matrix for cell type deconvolution.

**Computational Cell Type Deconvolution**

We used reference-based cell type deconvolution to estimate cellular proportions using Scaden (version 1.1.2), with default settings. This technique trains a deep neural network on simulated bulk RNA-seq data derived generated from the scRNA-seq reference dataset. We used 2 reference datasets (1) scRNA datsaet from this study and (2) reference of Tabula Sapiens (10 major cell types). To address the variations in the estimated cell type proportions between runs, the deconvolution was repeated 10 times for each pair. The average of these runs was taken for further analysis.

**Association of cell proportions with symptoms and major adverse cardiovascular event risk**

Normalized gene expression values were standardized to a mean of 0 and SD of 1 (scaled to z-score) before the analysis. As previously described, symptom severity was categorized as mild (asymptomatic or ocular complications) or severe (TIA or stroke). The association between symptom severity and the cell type proportions was analysed using a general linear model. To evaluate the relationship between cell type proportions and the risk of future MACE over a three-year period, Cox regression analyses were conducted. Cox regression models were adjusted for known risk factors of MACE, including age, sex, body mass index (BMI), HDL cholesterol levels, glomerular filtration rate, peripheral arterial occlusive disease (PAD), diabetes and the history of coronary artery disease. The missing clinical datapoints were imputed using mice R package.

To validate the association with future MACE, a Cox regression analysis was performed on the standardized gene expression data from 82 human carotid plaques from the CPIP cohort, adjusted for age, sex, BMI, PAD, diabetes and history of heart disease. A detailed description of the RNA sequencing analysis, including quality control and data processing, has previously been published(21, 22). During the follow-up, up to 10 years until December 2015, 13 out of the 82 patients suffered from a MACE.

Hazard ratios (HRs) per 1 standard-deviation (SD) increase, with 95% confidence intervals (CIs), were reported from both analyses.

**Evaluation of model discrimination with gene expression modules**

To assess the incremental predictive value of cell-type-specific gene expression, logistic regression models were constructed with occurrence of MACE within three years as outcome. A base model including classical risk factors (age, sex, BMI, HDL cholesterol, glomerular filtration rate, PAD, diabetes and history of coronary artery disease) was used as reference. To this base model, standardized expression values of individual genes belonging to predefined signatures were added. Separate models were tested for lipid-associated macrophage (LAM), inflammatory LAM (iLAM), resident macrophage, and inflammatory macrophage gene sets.

Model discrimination was evaluated by calculating the area under the receiver operating characteristic curve (AUC). Improvements in discrimination relative to the base model were quantified as ΔAUC, and statistical significance was assessed using the DeLong test implemented in the pROC R package.

### Primary monocyte isolation and culture

Peripheral blood mononuclear cells (PBMCs) were isolated from buffy coats obtained from healthy volunteers via Sanquin blood bank (Amsterdam, The Netherlands). All subjects provided written informed consent before donation. Whole blood was diluted in phosphate-buffered saline (PBS; Sigma-Aldrich, cat#D8537) and layered over Lymphoprep (Axis-Shield, cat#1114545) density gradient separation medium. The PBMC interlayer was collected and washed twice with cold PBS, and erythrocytes were lysed using red blood cell lysis buffer (eBioscience, cat#00433357). CD14^+^ and CD16^+^ human monocytes were isolated using Miltneyi Biotec CD14^+^ and CD16^+^ monocyte isolation kits, respectively (Milteny, cat#130050201). Isolated monocytes were either cultured in the presence of 50 ng/ml M-CSF (Miltenyi, cat#130096493) or GM-CSF (Miltenyi, cat#130095372) for 6 days to differentiate into macrophages or incubated with DMSO or 10 µM Firategrast for 1 hour before the flow experiments. Cells were cultured in Gibco IMDM medium (Thermo Fisher Scientific, cat#21056023) supplemented with 10% filtered Gibco Fetal Bovine Serum (FBS), qualified, heat-inactivated (Thermo Fisher Scientific, cat#10500064), 2mM L-glutamine (Thermo Fisher Scientific, cat#25030024), 100U/mL Gibco penicillin, 100µg/mL Gubco streptomycin (Thermo Fischer Scientific, cat#15140122) at 37°C and 5% CO_2_ in a humified atmosphere.

Monocyte-derived macrophages were incubated with 10 ng/ml LPS Sigma-Aldrich, E. coli 055:B5 cat#L2637) or 50 μg/ml acetylated LDL (acLDL) (Kalen Biomedical, cat#770201) alone or in combination with 50 μg/ml oxidized LDL (oxLDL) for 24 hours. After this RNA was isolated using the Qiagen RNAeasy kit, or intracellular lipids were stained with Oil red O (ORO).

**ORO staining:** Ac-LDL stimulated cells were plated on Nunc® Lab-Tek® Chamber Slide systems (Thermo Fischer Scientific, cat#155361). After washing the cells twice with PBS, they were fixed using 4% formalin for 10 minutes, then washed twice in 60% isopropanol for 15 minutes, and stained with 0.3% ORO staining (Sigma-Aldrich, cat#O0625) in 60% isopropanol and 39,7% demi water for 45 minutes. Excess stain was removed by dipping in 60% isopropanol, after which cells were washed in PBS, and nuclei were stained by dipping in 33% heamatoxylin (Sigma-Aldrich, cat#H3136) in demi water. Intracellular lipid accumulation was captured using a Leica DM3000 microscope at 20x magnification. Images were analyzed with QuPath, and ORO-positive pixels were quantified using R scripts.

**rt-qPCR:** RNA from cells was isolated using the RNeasy mini kit (Qiagen, cat#74104), followed by synthesis of cDNA libraries using the high-capacity cDNA reverse transcription kit (Thermo Fisher Scientific, cat#4368813). qPCR was performed using the fast SYBR Green method (Thermo Fischer Scientific, cat#A25742). qPCR reactions were ran on a Thermo Fisher QuantStudio 5 machine following manufacturer’s instructions. Results were assessed for relative mRNA expression and normalized to two housekeeping genes (HPRT1 and GNB2L1), designated stimuli, and vehicle controls using the 2^-ΔΔCt^ method. Primer sequences are supplied in supplementary table 1.

### Immuno Histo Chemistry:

Human atherosclerotic plaque samples were obtained from carotid endarterectomy surgery at Maastricht University Hospital and pieces of 5 mm thickness were snap frozen in Optimal Cutting Temperature (OCT) compound and stored at -80° C. Tissue sections were prepared, stored and stained as previously described(23). In short, 7 µm thick sections were mounted on standard microscopy slides (VWR), dried in a desiccator at RT overnight and stored at -80 °C. Prior to staining, they were thawed, fixed with dry acetone for 5 minutes and gently rinsed with Phosphate-buffered saline (PBS) before being immersed in blocking buffer (4% Fetal Calf Serum (FCS) in PBS (0.2 µm-filtered)) for 3h bleaching by high-intensity white LED light at 4 °C. Sections were incubated with a panel of antibodies and a 7-AAD nuclear stain (1:100, BD Biosciences) in PBS + 4% FCS for 3 h at RT and washed 3 times with PBS. Coverslips were mounted using Prolong Gold (ThermoFisher) and curated overnight at RT. Multispectral imaging was performed on a fluorescence microscope (Leica DM4000) equipped with a Nuance FX camera (Perkin Elmer), as previously described(23, 24). Dye-specific spectra and autofluorescence were defined on unstained and single-marker stained sections and saved in a spectral library, which was used to unmix the images taken of samples stained with the entire panel into their individual spectral components using the Nuance software (PerkinElmer). The resulting images representing the individual antibodies’ signals were corrected for background signal and combined into a composite image using Fiji(25).

### Flow Cytometry

Atherosclerotic plaque samples were collected and processed from 3 donors undergoing endarterectomy surgery at Haaglanden Medical Center (The Hague) as previously described(26). Single cell suspensions stored in cryostor (-150^o^C) were thawed at 37^o^C, in RPMI (Gibco, 52400-025) containing 10% FCS (Gibco A5256801), and incubated at 37 ^o^C with DNAse I (Roche, 04536282001). Samples were stained with viability dye (Supplemental Resource Table), following staining with the extracellular antibody mix (Supplemental Resource Table), fixation (Invitrogen, 00-5523-00) and sequential incubation with the intracellular antibody mix. Both antibody mixtures contained Human TruStain FcX™ (Biolegend, 422302), True-Stain Monocyte Blocker™ (Biolegend, 426103), and BD Horizon™ Brilliant Stain Buffer (BD, 659611). Samples were acquired in the ID7000™ Spectral Cell Analyzer (320nm, 355nm, 405nm, 488nm, 561nm, 637nm), Sony Biotechnology. Autofluorescence correction, and unmixing was performed in the ID7000™ Software (Version 2.2.1.17271). The OMIQ software was used to generate figures as well as for sample gating, UMAP, and FlowSOM analysis.

## References

1. Depuydt MAC, Prange KHM, Slenders L, Ord T, Elbersen D, Boltjes A, et al. Microanatomy of the Human Atherosclerotic Plaque by Single-Cell Transcriptomics. Circ Res. 2020;127(11):1437-55.

2. Depuydt MAC, Schaftenaar FH, Prange KHM, Boltjes A, Hemme E, Delfos L, et al. Single-cell T cell receptor sequencing of paired human atherosclerotic plaques and blood reveals autoimmune-like features of expanded effector T cells. Nature Cardiovascular Research. 2023;2(2):112-25.

3. Hao Y, Hao S, Andersen-Nissen E, Mauck WM, 3rd, Zheng S, Butler A, et al. Integrated analysis of multimodal single-cell data. Cell. 2021;184(13):3573-87 e29.

4. Luecken MD, Buttner M, Chaichoompu K, Danese A, Interlandi M, Mueller MF, et al. Benchmarking atlas-level data integration in single-cell genomics. Nat Methods. 2022;19(1):41-50.

5. Wickham H. ggplot2: Elegant Graphics for Data Analysis. New York: Springer-Verlag New York; 2016.

6. Kassambara A. ggpubr: 'ggplot2' Based Publication Ready Plots 2023 [Available from: <https://rpkgs.datanovia.com/ggpubr/>.

7. Menden K, Marouf M, Oller S, Dalmia A, Magruder DS, Kloiber K, et al. Deep learning-based cell composition analysis from tissue expression profiles. Sci Adv. 2020;6(30):eaba2619.

8. Alhamdoosh M, Ng M, Wilson NJ, Sheridan JM, Huynh H, Wilson MJ, et al. Combining multiple tools outperforms individual methods in gene set enrichment analyses. bioRxiv. 2016:042580.

9. Badia IMP, Velez Santiago J, Braunger J, Geiss C, Dimitrov D, Muller-Dott S, et al. decoupleR: ensemble of computational methods to infer biological activities from omics data. Bioinform Adv. 2022;2(1):vbac016.

10. Cao J, Packer JS, Ramani V, Cusanovich DA, Huynh C, Daza R, et al. Comprehensive single-cell transcriptional profiling of a multicellular organism. Science (New York, NY). 2017;357(6352):661-7.

11. Trapnell C, Cacchiarelli D, Grimsby J, Pokharel P, Li S, Morse M, et al. The dynamics and regulators of cell fate decisions are revealed by pseudotemporal ordering of single cells. Nat Biotechnol. 2014;32(4):381-6.

12. La Manno G, Soldatov R, Zeisel A, Braun E, Hochgerner H, Petukhov V, et al. RNA velocity of single cells. Nature. 2018;560(7719):494-8.

13. Lange M, Bergen V, Klein M, Setty M, Reuter B, Bakhti M, et al. CellRank for directed single-cell fate mapping. Nat Methods. 2022;19(2):159-70.

14. Love MI, Huber W, Anders S. Moderated estimation of fold change and dispersion for RNA-seq data with DESeq2. Genome Biology. 2014;15(12):550.

15. Hanzelmann S, Castelo R, Guinney J. GSVA: gene set variation analysis for microarray and RNA-seq data. BMC Bioinformatics. 2013;14:7.

16. Freshour SL, Kiwala S, Cotto KC, Coffman AC, McMichael JF, Song JJ, et al. Integration of the Drug-Gene Interaction Database (DGIdb 4.0) with open crowdsource efforts. Nucleic Acids Res. 2021;49(D1):D1144-D51.

17. Buttner M, Miao Z, Wolf FA, Teichmann SA, Theis FJ. A test metric for assessing single-cell RNA-seq batch correction. Nat Methods. 2019;16(1):43-9.

18. Korsunsky I, Millard N, Fan J, Slowikowski K, Zhang F, Wei K, et al. Fast, sensitive and accurate integration of single-cell data with Harmony. Nat Methods. 2019;16(12):1289-96.

19. Stuart T, Butler A, Hoffman P, Hafemeister C, Papalexi E, Mauck WM, 3rd, et al. Comprehensive Integration of Single-Cell Data. Cell. 2019;177(7):1888-902 e21.

20. Reuter B, Weber M, Fackeldey K, Roblitz S, Garcia ME. Generalized Markov State Modeling Method for Nonequilibrium Biomolecular Dynamics: Exemplified on Amyloid beta Conformational Dynamics Driven by an Oscillating Electric Field. J Chem Theory Comput. 2018;14(7):3579-94.

21. Sun J, Singh P, Shami A, Kluza E, Pan M, Djordjevic D, et al. Spatial Transcriptional Mapping Reveals Site-Specific Pathways Underlying Human Atherosclerotic Plaque Rupture. J Am Coll Cardiol. 2023;81(23):2213-27.

22. Goncalves I, Oduor L, Matthes F, Rakem N, Meryn J, Skenteris NT, et al. Osteomodulin Gene Expression Is Associated With Plaque Calcification, Stability, and Fewer Cardiovascular Events in the CPIP Cohort. Stroke. 2022;53(3):e79-e84.

23. Wieland EB, Kempen L, Lu C, Donners M, Biessen EAL, Goossens P. Protocol for multispectral imaging on cryosections to map myeloid cell heterogeneity in its spatial context. STAR Protoc. 2023;4(4):102601.

24. Goossens P, Lu C, Cao J, Gijbels MJ, Karel JMH, Wijnands E, et al. Integrating multiplex immunofluorescent and mass spectrometry imaging to map myeloid heterogeneity in its metabolic and cellular context. Cell Metab. 2022;34(8):1214-25 e6.

25. Schindelin J, Arganda-Carreras I, Frise E, Kaynig V, Longair M, Pietzsch T, et al. Fiji: an open-source platform for biological-image analysis. Nat Methods. 2012;9(7):676-82.

26. Kritikou E, Depuydt MAC, de Vries MR, Mulder KE, Govaert AM, Smit MD, et al. Flow Cytometry-Based Characterization of Mast Cells in Human Atherosclerosis. Cells. 2019;8(4).
